# Synergistic toxicity and clinical prioritization of docetaxel combined with nintedanib for non-small cell lung cancer: a real-world safety analysis using the FAERS database

**DOI:** 10.3389/fonc.2026.1836198

**Published:** 2026-07-27

**Authors:** Qian Zhang, Qinqin Feng, Shuang Lei, Wei Yang, Wanpeng Zhang, Xifeng Liu

**Affiliations:** 1 Department of Pharmacy, Norinco General Hospital, Xi’an, Shaanxi, China; 2 The Center for Drug Safety and Policy Research, Xi’an Jiaotong University, Xi’an, Shaanxi, China; 3 Zhuoxunda Technology Development Co., Ltd., Shenzhen, Guangdong, China; 4 Xi’an Rail Transit Group Co., Ltd. Operations Branch, Xi’an, Shaanxi, China

**Keywords:** adverse drug reactions/events, clinical prioritization, disproportionality analysis, FAERS, non-small cell lung cancer

## Abstract

**Background:**

Non-small cell lung cancer (NSCLC) accounts for 85% of new lung cancer diagnoses. Although docetaxel–nintedanib combination therapy improves survival in previously treated NSCLC, its real-world safety profile has not been comprehensively characterized. This study aimed to comprehensively characterize the real-world safety profile of docetaxel, nintedanib, and their combination in patients with NSCLC.

**Methods:**

We retrieved adverse drug reaction(ADR) reports associated with docetaxel, nintedanib monotherapy and their combination in NSCLC patients from the FAERS database. Disproportionality analyses using the Reporting Odds Ratio (ROR) and Bayesian Confidence Propagation Neural Network (BCPNN) were performed, followed by clinical priority scoring and time-to-onset analyses.

**Results:**

A total of 54, 66, and 78 significant PT-level safety signals were identified for docetaxel monotherapy, nintedanib monotherapy, and their combination therapy, respectively, and all signals remained significant after BH-FDR correction (P<0.05). Compared with either monotherapy, the combination regimen demonstrated a broader toxicity spectrum with relatively greater reporting of hematologic, gastrointestinal, infectious, and hepatobiliary adverse reactions. Combination therapy showed disproportionately strong signals for mucosal infection (ROR = 343.76, IC = 8.40, p<0.001), metastases to adrenals (ROR = 90.15, IC = 6.49, p<0.001) and portal vein thrombosis (ROR = 28.43, IC = 4.83, p<0.001). Docetaxel monotherapy was predominantly associated with skin and psychiatric disorders, whereas nintedanib monotherapy was predominantly associated with hepatic injury and thromboembolic events. Clinical prioritization identified febrile neutropenia, intestinal perforation, and sepsis as the highest-priority adverse events in the combination group. Median time-to-onset was shorter for combination therapy than for docetaxel or nintedanib monotherapy (22.5 vs. 34.5 and 34.5 days, Log-rank p<0.001). Weibull analysis indicated an early-failure pattern (β < 1) for all three regimens.

**Conclusion:**

This study provides a comprehensive real-world comparison of the safety profiles of docetaxel, nintedanib, and their combination, identifying distinct toxicity patterns and clinically important adverse reactions. These findings may facilitate individualized safety monitoring and optimize toxicity management in patients with NSCLC.

## 1 Background

Lung cancer remains the leading cause of cancer-related mortality worldwide, with non-small cell lung cancer (NSCLC) accounting for approximately 85% of all new cases (1). NSCLC is broadly classified into squamous cell carcinoma and non-squamous cell carcinoma, the latter encompassing histological subtypes such as adenocarcinoma and large cell carcinoma. Due to the absence of specific symptoms in early stages, approximately 57% of patients are diagnosed at locally advanced or metastatic stages, posing substantial challenges to public health systems. Although first-line platinum-based chemotherapy, targeted therapies, and immunotherapies have markedly improved survival outcomes in selected populations, most patients experience disease progression within one year following completion of first-line sequential therapy. Consequently, effective and well-tolerated second-line treatment options remain limited (2).

Docetaxel, as a second- or later-line therapeutic agent for advanced NSCLC, represents one of the established standard treatments. However, its modest survival benefit and limited median overall survival have constrained its long-term clinical utility. Given that the tumor microenvironment harbors multiple pro-angiogenic factors, angiogenesis plays a pivotal role in tumor growth and metastasis, positioning anti-angiogenic strategies as an integral component of NSCLC management (3, 4). Nintedanib, an oral multi-target tyrosine kinase inhibitor, exerts anti-angiogenic and antitumor effects by inhibiting key signaling pathways, including vascular endothelial growth factor receptors (VEGFRs), platelet-derived growth factor receptors (PDGFRs), and fibroblast growth factor receptors (FGFRs) (5). The combination of nintedanib with docetaxel operates through a “two-way street” mechanism (6): docetaxel directly eliminates tumor cells and disrupts microtubule function, while nintedanib concurrently inhibits tumor angiogenesis and suppresses pathways involved in tumor cell proliferation and migration. This dual action creates a potential synergistic antitumor effect (7, 8). The pivotal LUME-Lung 1 trial demonstrated that this combination significantly prolonged median progression-free survival in patients with adenocarcinoma histology compared to docetaxel plus placebo (3.4 months vs. 2.7 months; P = 0.0019) (9). Furthermore, a study investigating nintedanib plus docetaxel in patients with advanced lung adenocarcinoma who had progressed following prior chemotherapy and immunotherapy reported a median overall survival of 8.8 months and a disease control rate of 78.2% (10). Based on accumulating clinical trial evidence, the combination of nintedanib and docetaxel has been formally approved in Europe and multiple other countries as second-line therapy for advanced non-squamous NSCLC, including adenocarcinoma and locally advanced, metastatic, or locally recurrent disease (11).

However, the survival benefits associated with combination therapy are accompanied by potential risks of synergistic toxicities (12). Current data on ADR related to nintedanib plus docetaxel derive predominantly from clinical trials. Evidence regarding the efficacy and safety of currently approved second-line agents following immunotherapy failure remains limited (13). A multicenter retrospective real-world study indicated that the occurrence of ADR was correlated with treatment outcomes in patients receiving this combination; notably, patients without adverse events had a median survival of two months, whereas those experiencing adverse events achieved a median survival of six months (14). Of particular concern, anti-angiogenic agents inherently carry risks of mucosal injury and hemorrhage, and their combination with chemotherapeutic agents may potentiate cumulative bone marrow suppression and organ toxicity (15–17). In the LUME-Lung 1 trial, over 60% of patients experienced grade ≥3 adverse events, including severe neutropenia, diarrhea, and elevated liver enzymes. Additionally, the non-interventional LUME-BioNIS study examining nintedanib plus docetaxel following chemotherapy in patients with adenocarcinoma NSCLC reported that 60.0% of patients experienced events considered related to the combination therapy, with diarrhea, nausea, vomiting, and malignant neoplasm progression being the most frequently observed ADR (18). Clinical trials are inherently constrained by limited sample sizes, relatively short follow-up durations, and stringent inclusion/exclusion criteria, thereby compromising their capacity to identify rare, delayed-onset, or population-specific adverse events encountered in real-world clinical practice.

Pharmacovigilance studies leveraging real-world data can effectively address these limitations by integrating large-scale safety information derived from actual clinical use. The U.S. Food and Drug Administration Adverse Event Reporting System (FAERS) constitutes a comprehensive repository of adverse event information originating from clinical practice, encompassing reports submitted both within the United States and internationally. This database not only captures rare and long-latency ADR but also provides a unique resource for investigating drug-drug interactions and identifying novel safety signals associated with combination regimens (19, 20), thereby facilitating comprehensive evaluation of the adverse reaction profiles of combination therapies. The present study aims to systematically evaluate the long-term safety profile of docetaxel combined with nintedanib in NSCLC patients within a real-world setting, characterize the common and distinctive features of adverse events under specific clinical circumstances, and identify critical safety signals and early-onset high-risk events associated with this combination regimen. These findings are intended to promote the safe and rational use of this therapeutic approach and provide evidence-based guidance for optimizing clinical risk monitoring strategies.

## 2 Methods

### 2.1 Data source and processing

This study was conducted in accordance with the READUS-PV guidelines to ensure the transparency and reproducibility of disproportionality analyses (21). We extracted reports from the FAERS database (https://fis.fda.gov/extensions/FPD-QDE-FAERS/FPD-QDE-FAERS.html) where docetaxel was recorded as the “Primary Suspect” (PS), covering the period from the first quarter (Q1) of 2014 to Q1 of 2025. Additionally, we retrieved ADR/E reports involving the combined use of nintedanib and docetaxel for NSCLC patients, records in the DRUG table where both docetaxel (PS) and nintedanib (PS or Secondary Suspect [SS]) were present.

The FAERS database contains various adverse event reports from healthcare professionals, consumers, and other sources. Visitors can directly download quarterly data files from the database, which include seven standard data tables: DEMO, DRUG, REAC, THER, INDI, OUTC, and others (22). Following FDA guidance, we handled duplicate PRIMARY_IDs by prioritizing the record with the latest FDA_DT. In cases where FDA_DT and CASE_ID matched exactly, the record possessing the higher PRIMARY_ID value was chosen to filter out redundant submissions (23, 24). For records with consistent FDA_DT and CASE_ID, we reserved the entry with the maximum PRIMARY_ID to remove redundant reports submitted via different channels for one single adverse event occurrence. Furthermore, we used the Medical Dictionary for Regulatory Activities (MedDRA) to classify ADR into System Organ Classes (SOCs) and Preferred Terms (PTs) (25). Due to the arbitrary listing of drugs in the FAERS database, we used MICROMEDEX as a reference source and applied all generic names, brand names, and clinical trial codes. In addition, we excluded reports with missing drug names, missing adverse events, or ADR unrelated to the study drugs. Records with missing age or gender data were retained in the overall analysis but excluded from the corresponding subgroup analyses.

### 2.2 Signal detection and statistical analysis

Real-world pharmacovigilance studies based on the FAERS database require data mining and multi-dimensional evaluation to assess the safety of nintedanib combined with chemotherapy. As an important tool for monitoring and evaluating drug safety, disproportionality analysis can identify potential risk signals from the large volume of adverse event information in the FAERS database (26). Its basic assumption is that a potential association exists between a target drug and an adverse event when the frequency of the adverse event in the population using the target drug is significantly higher than that in the population not using the target drug, and this difference exceeds a predefined threshold. Various disproportionality metrics are available, including Bayesian and frequentist methods (27). To improve the reliability of results and avoid interference from false-positive signals, we used the Reporting Odds Ratio (ROR) and Bayesian Confidence Propagation Neural Network (BCPNN) for ADR/E signal detection. The combined use of these two methods integrates the efficiency of frequentist statistics and the robustness of Bayesian methods (28, 29). Detailed calculation formulas and statistical thresholds are provided in the **Supplementary Materials** (**Supplementary Table 1**). Following PT-level signal detection, events primarily reflecting disease progression or lack of therapeutic efficacy (e.g., malignant neoplasm progression and related progression terms) were excluded from subsequent analyses to minimize confounding arising from the natural history of the underlying malignancy.

To further evaluate the statistical robustness of PT-level safety signals identified via predefined ROR and BCPNN criteria, we conducted supplementary validation analyses incorporating both Bonferroni correction and Benjamini–Hochberg false discovery rate (BH-FDR) adjustment. The Bonferroni-adjusted significance threshold was defined as Padj = Praw × m (Padj < 0.05), where m denotes the number of PT-level signals included in the validation analysis. In parallel, the Benjamini–Hochberg false discovery rate (BH-FDR) procedure was applied to control the expected proportion of false discoveries, using an FDR threshold of 0.05.

Descriptive statistics were used to analyze the demographic information related to nintedanib combined with chemotherapy. Meanwhile, the age- and sex-specific analyses were conducted as sensitivity analyses to assess the robustness and demographic heterogeneity of the identified ADR signals. We used odds ratios (ORs) to quantify risk differences for common ADRs according to gender (male and female) and patient age (divided into 18–64 years and ≥ 65 years) to explore the similarities and differences in signals among subgroups with different characteristics. ORs were calculated using 2×2 contingency tables without multivariable adjustment.

In addition, to evaluate the potential influence of reporting-source heterogeneity on signal detection, a sensitivity analysis was performed by excluding all lawyer-submitted reports from the FAERS dataset. Disproportionality analyses were subsequently repeated using the same signal detection criteria as in the primary analysis. The consistency of signal strength before and after exclusion was assessed using Spearman’s rank correlation coefficient. Then, the overlap of detected signals between the primary and sensitivity analyses was compared to evaluate the robustness of the identified safety signals.

### 2.3 Clinical priority assessment of ADR signals

All significant signals underwent a structured clinical priority assessment based on a multi-parameter scoring system. Each PT was evaluated and scored across the following four domains (30). Detailed assessment criteria were provided in the **Supplementary Materials** (**Supplementary Table 2**).

1. Clinical relevance: Using the list of Important Medical Events (IMEs) and Designated Medical Events (DMEs) provided by European medical institutions.
2. Reporting rate: The ratio of target ADR to non-target ADR. Reporting rates were categorized into the following traditional categories: very common (≥ 10%), common (1-10%), and uncommon (≤ 1%).
3. Signal stability: Based on the consistency of results from multiple signal detection methods.
4. Reported mortality: The severity was evaluated by calculating the case fatality rate, while considering a conservative strategy for assessment in oncology and other fields, where the highest score was assigned only when the case fatality rate exceeded 50%.

It’s important to note that in oncology it’s difficult to distinguish drug-induced adverse events from those due to natural disease progression. We adopted a conservative approach whereby the highest risk score was assigned only when drug-attributable fatality rates exceed 50%. A score of 0-2, 3-5, and 6–8 identified, respectively, AEs with low, moderate, or high priority.

### 2.4 Time-to-onset analysis

Time-to-Onset (TTO) was defined as the duration between the initiation of drug therapy (START_DT, sourced from the THER dataset) and the occurrence of an adverse event (EVENT_DT). This metric facilitates the characterization of the temporal distribution of ADR/E occurrences (31). Specifically, reports were excluded if they met any of the following criteria (1): missing EVENT_DT (2); missing START_DT (3); EVENT_DT occurring earlier than START_DT, resulting in a negative TTO value; or (4) incomplete, implausible, or obviously erroneous date records. Consequently, only reports containing complete and chronologically valid treatment initiation and adverse event onset dates were retained for TTO analysis.

Furthermore, following treatment initiation, the incidence of ADR was influenced by the drug’s mechanism of action and often varies over time. To investigate the temporal pattern of ADR occurrence following combination therapy, Weibull Shape Parameter (WSP) analysis was employed. The Weibull distribution is characterized by two parameters: the scale parameter (α), which reflects the time scale of ADR/E occurrence, and the shape parameter (β).The β<1 indicates that the incidence of ADR decreases over time; β=1 suggests no specific temporal clustering of ADR; and β>1 indicates that the incidence increases over time (32). The cumulative incidence of ADR was visualized using the Kaplan-Meier method, and differences in cumulative incidence curves between two treatment regimens and across subgroups were compared using the log-rank test.

We used R 4.3.3 software for statistical analysis and graph plotting. All programs were conducted in accordance with the FDA Signal Detection Methodology Guidance Document. All statistical tests were two-sided, and the confidence intervals of all estimates were at the 95% level. A p-value <0.05 was considered statistically significant.

## 3 Results

### 3.1 Descriptive analysis

After data preprocessing, including 8,341 reports for docetaxel monotherapy, 937 reports for nintedanib monotherapy, and 1,459 reports for the docetaxel–nintedanib combination therapy were extracted (**Table 1**). In the docetaxel monotherapy group, females accounted for the majority of reports (77.7%), whereas males predominated in both the nintedanib monotherapy group (45.3% vs. 35.8% female) and the combination therapy group (54.5% vs. 41.3% female). Most reports involved adult and elderly patients. Individuals aged 18–64 years represented the largest proportion in the docetaxel group (69.1%), while patients aged ≥65 years accounted for a relatively higher proportion in the nintedanib (37.8%) and combination therapy groups (43.7%). Healthcare professionals constituted the predominant reporters for nintedanib monotherapy (65.3%) and combination therapy (88.1%), whereas lawyer-submitted reports accounted for the majority of docetaxel monotherapy reports (61.4%). Regarding clinical outcomes, the proportion of death reports was highest in the nintedanib monotherapy group (34.3%), compared with 9.7% in the docetaxel group and 9.4% in the combination therapy group. Hospitalization was reported in 27.4% and 27.1% of reports in the nintedanib and combination therapy groups, respectively, whereas the corresponding proportion in the docetaxel group was 9.2%.

**Table 1:** Characteristics of adverse drug reaction reports associated with docetaxel, nintedanib and their combination from the FAERS database.

Characteristics	Reports, N (%)		
	Docetaxel	Nintedanib	Docetaxel+Nintedanib
Overall	N=8341	N=937	N=1459
Sex			
Female	6484 (77.7)	335 (35.8)	603 (41.3)
Male	1553 (18.5)	424 (45.3)	795 (54.5)
Unknown or missing	313 (3.8)	178 (19.0)	61 (4.2)
Weight, kg			
<50	105 (1.3)	74 (7.9)	47 (3.2)
50-100	2643 (31.7)	195 (20.8)	695 (47.6)
>100	423 (5.1)	9 (1.0)	21 (1.4)
Unknown or missing	5170 (62.0)	659 (70.3)	696 (47.7)
Age group, years			
<18	29 (0.3)	8 (0.9)	4 (0.3)
18-64	5762 (69.1)	226 (24.1)	709 (48.6)
≥65	2008 (24.1)	354 (37.8)	637 (43.7)
Unknown or missing	542 (6.5)	349 (37.2)	109 (7.5)
Reporter			
Health professionals	2126 (25.5)	612 (65.3)	1286 (88.1)
Pharmacists	126 (1.5)	91 (9.7)	22 (1.5)
Consumers	462 (5.5)	128 (13.7)	27 (1.9)
Lawyers	5124 (61.4)	0 (0)	0 (0)
Other health-professional	476 (5.7)	87 (9.3)	120 (8.2)
Unknown or missing	27 (0.4)	19 (2.0)	4 (0.3)
Outcome			
Death	807 (9.7)	321 (34.3)	80 (9.4)
Life-threatening	248(3.0)	14 (1.5)	15 (1.8)
Hospitalization (initial or prolonged)	768 (9.2)	257 (27.4)	230 (27.1)
Disability	39 (0.5)	2 (0.2)	1 (0.1)
Congenital Anomaly	3 (0.1)	0 (0)	0 (0)
Other	6483 (77.5)	343 (36.6)	162 (19.1)
Reporting country			
Europe	1550 (19.5)	212 (24.2)	1317 (90.3)
Asia	559 (7.0)	372 (42.5)	90 (6.1)
North America	5827 (73.3)	282 (32.2)	5 (0.4)
Other	10 (0.2)	10 (1.1)	47 (3.2)

### 3.2 Signal detects at the SOC level

After excluding non-drug-related events such as product issues and social circumstances, we screened common or important SOCs in both groups based on the number of events and disproportionality analysis (lower limit of ROR 95% CI > 1 or IC025 > 0) (**Supplementary Tables 3**–**5**).

For docetaxel monotherapy, the most frequently reported SOCs were skin and subcutaneous tissue disorders, general disorders and administration site conditions, and psychiatric disorders; the former two categories yielded significant positive disproportionality signals. For nintedanib monotherapy, top-reported SOCs included gastrointestinal disorders and general disorders and administration site conditions, with gastrointestinal disorders, neoplasms benign, malignant and unspecified, blood and lymphatic system disorders showing significant signals. For combination therapy, the most common SOCs were gastrointestinal disorders, general disorders and administration site conditions, and neoplasms benign, malignant and unspecified, all three of which presented positive disproportionality signals.

**Figure 1:**
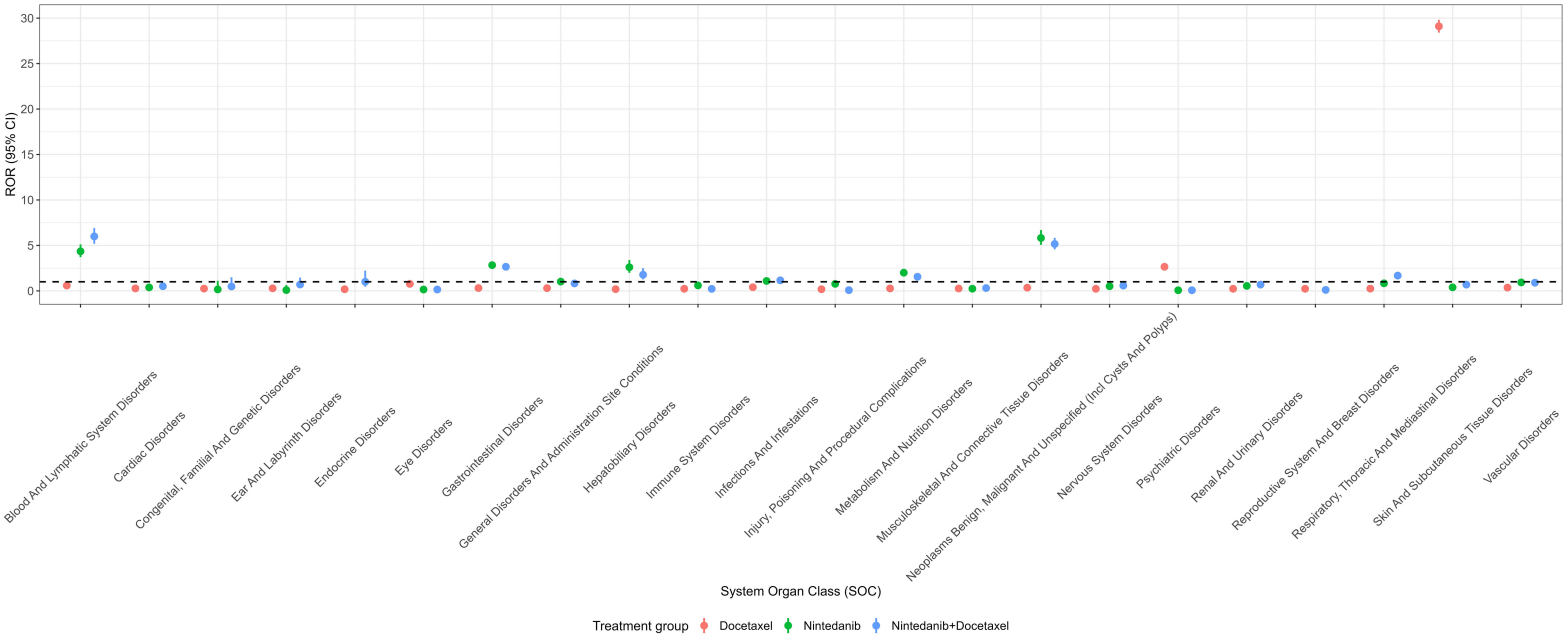
Comparison of adverse drug reactions at the SOC level among docetaxel, nintedanib and their combination. SOC, system organ class; DTX, docetaxel; NinoDoc, docetaxel-nintedanib combination therapy; ROR, reporting odds ratio; 95%CI, 95% confidence interval.

As shown in **Figure 1**, the results demonstrated that the combination group exhibited substantially stronger overall ADR signals than both monotherapy arms, with statistically significantly elevated risks of ADRs across multiple organ systems such as blood and lymphatic system, gastrointestinal tract, metabolism and nutrition, as well as respiratory and mediastinal disorders. Single-agent docetaxel was associated with remarkably higher risks of skin and subcutaneous tissue disorders, with a far greater ROR value for this SOC compared with single-agent nintedanib and the combination regimen. In contrast, single-agent nintedanib predominantly induced characteristic adverse events including metabolic and electrolyte disturbances, musculoskeletal pain, and asthenia.

### 3.3 ADR signal detection at the PT level

Based on combined ROR and IC criteria, 54, 65 and 78 positive PT signals were identified for docetaxel, nintedanib and their combination therapy, respectively. All signals from the three groups passed the BH-FDR correction. After stricter Bonferroni correction, 45 signals remained significant for docetaxel, 51 for nintedanib, and 65 for their combination (**Supplementary Tables 6**–**8**).

For combination therapy, the top three ADRs with the strongest associations were mucosal infection (ROR = 343.76, IC = 8.40, p<0.001), metastases to adrenals (ROR = 90.15, IC = 6.49, p<0.001) and portal vein thrombosis (ROR = 28.43, IC = 4.83, p<0.001), as detailed in **Supplementary Table 7** and **Table 2**. For docetaxel monotherapy, the top three ADRs with the strongest associations were psychological trauma (ROR = 3976.4, IC = 4.36, p<0.001), madarosis (ROR = 1648.57, IC = 4.34, p<0.001), and hair disorder (ROR = 1648.57, IC = 4.34, p<0.001), as detailed in **Supplementary Table 6**. For nintedanib monotherapy, the top three ADRs with the strongest associations were cholangitis infective (ROR = 448.92, IC = 8.71 p<0.001), biliary tract infection (ROR = 86.15, IC = 6.41, p<0.001), and duodenal stenosis (ROR = 172.3, IC = 7.39, p<0.001), as detailed in **Supplementary Table 8**.

**Table 2:** Top 20 signal strengths of the docetaxel–nintedanib combination at the preferred term level.

Preferred term (PT)	Cases (n)	ROR (95%CI)	IC (IC025)	Bonferroni_p	BH-FDR
Mucosal infection	3	343.76 (109.66-1077.56)	8.40 (0.53)	p<0.001	P<0.001
Metastases to adrenals	3	90.15 (28.97-280.47)	6.49 (0.50)	p<0.001	p<0.001
Metastases to meninges	4	46.64 (17.47-124.52)	5.54 (0.91)	p<0.001	p<0.001
Metastases to central nervous system	18	38.24 (24.04-60.83)	5.24 (3.02)	p<0.001	p<0.001
Idiopathic pulmonary fibrosis	6	37.27 (16.71-83.11)	5.21 (1.50)	p<0.001	p<0.001
Metastases to peritoneum	3	31.23 (10.06-97.00)	4.96 (0.42)	p<0.001	p<0.001
Portal vein thrombosis	3	28.43 (9.16-88.29)	4.83 (0.41)	p<0.001	p<0.001
Peripheral sensory neuropathy	6	28.27 (12.68-63.03)	4.82 (1.44)	p<0.001	p<0.001
Neutropenic sepsis	7	27.73 (13.20-58.27)	4.79 (1.65)	p<0.001	p<0.001
Polyneuropathy	12	26.77 (15.18-47.23)	4.73 (2.36)	p<0.001	p<0.001
Febrile neutropenia	55	22.98 (17.59-30.04)	4.49 (3.63)	p<0.001	p<0.001
Gastrointestinal toxicity	4	22.88 (8.57-61.04)	4.51 (0.80)	p<0.001	p<0.001
Metastases to lung	9	20.66 (10.73-39.77)	4.36 (1.88)	p<0.001	p<0.001
Metastases to lymph nodes	5	20.29 (8.43-48.83)	4.34 (1.09)	p<0.001	p<0.001
Hepatic lesion	3	19.17 (6.17-59.51)	4.26 (0.34)	p<0.001	p<0.001
Metastases to liver	12	18.43 (10.45-32.52)	4.20 (2.17)	p<0.001	p<0.001
Mucosal inflammation	16	17.55 (10.73-28.70)	4.12 (2.44)	p<0.001	p<0.001
Hematotoxicity	6	17.39 (7.80-38.75)	4.12 (1.29)	p<0.001	p<0.001
Hiccups	4	14.64 (5.49-39.07)	3.87 (0.68)	p<0.001	p<0.001
Gamma-glutamyltransferase increased	9	14.58 (7.58-28.07)	3.86 (1.71)	p<0.001	p<0.001

ROR, reporting odds ratio; 95%CI, 95% confidence interval; IC, information component; IC_025_: 95% confidence interval of IC; BH-FDR: the Benjamini–Hochberg false discovery rate.

To directly compare the ADR risks between the monotherapy and combination therapy groups, we used the OR method to quantify the risk differences between the two treatment regimens for common ADRs. Confidence intervals to the right of the null line (ROR = 1) indicate that the risk of the corresponding ADR was higher in the monotherapy group than in the combination therapy group.

**Figure 2:**
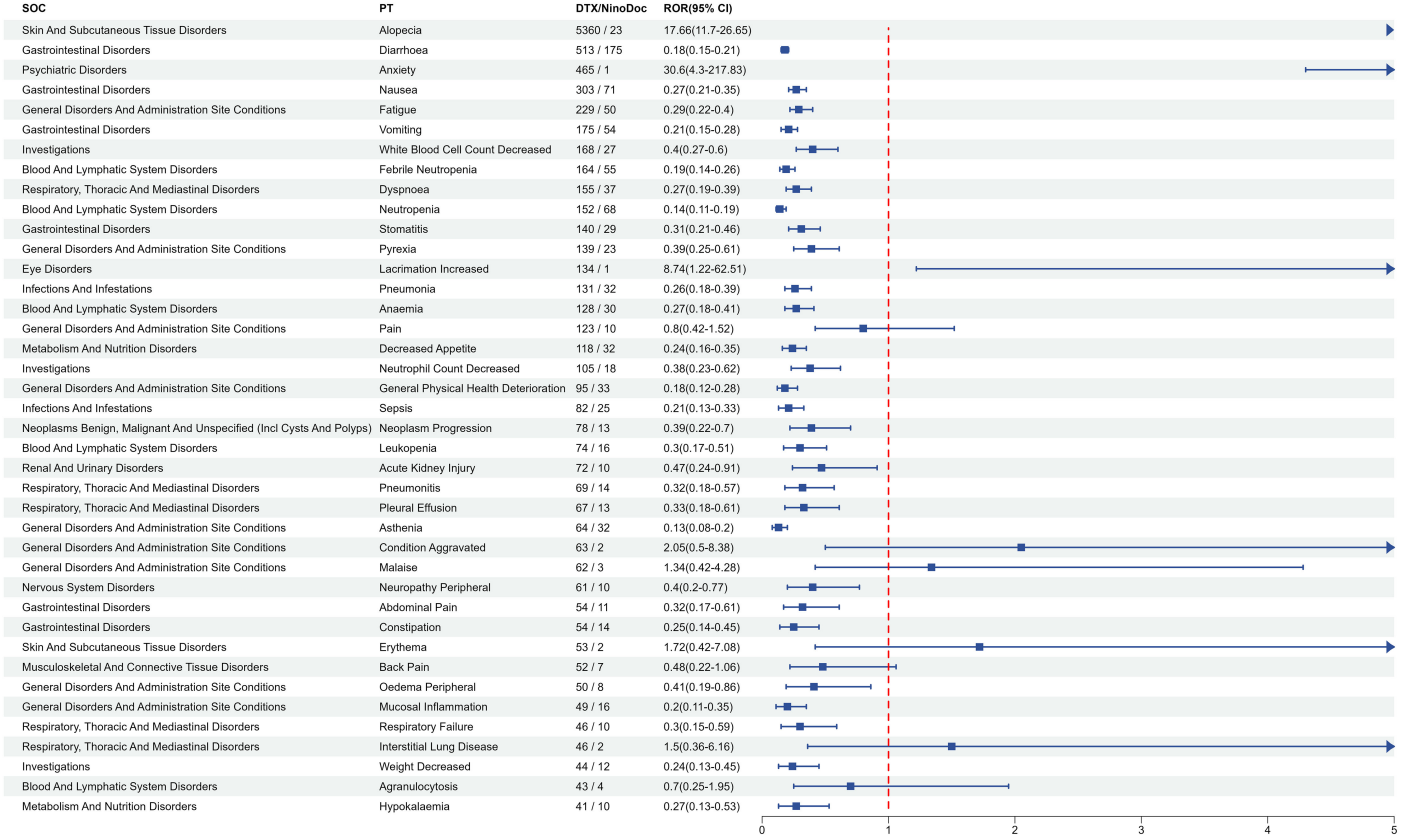
Comparison of AE signals between docetaxel monotherapy and docetaxel-nintedanib combination therapy at the PT level. SOC, system organ class; PT, Preferred term; DTX, docetaxel; NinoDoc, docetaxel-nintedanib combination therapy; ROR, reporting odds ratio; 95%CI, 95% confidence interval.

Compared with docetaxel monotherapy, the combination regimen demonstrated markedly higher reporting frequencies of hematologic toxicities, including febrile neutropenia, neutropenia, leukopenia, and decreased white blood cell count. Similarly, several gastrointestinal ADRs were more frequently reported in the combination group, including diarrhea, nausea, vomiting, stomatitis and abdominal pain. In addition, infection-related events such as sepsis and pneumonia were disproportionately enriched in the combination therapy group. In contrast, the docetaxel monotherapy group was mainly associated with skin appendage damage and psychosocial-related adverse reactions, including alopecia, anxiety, increased lacrimation, erythema, as shown in **Figure 2**.

**Figure 3:**
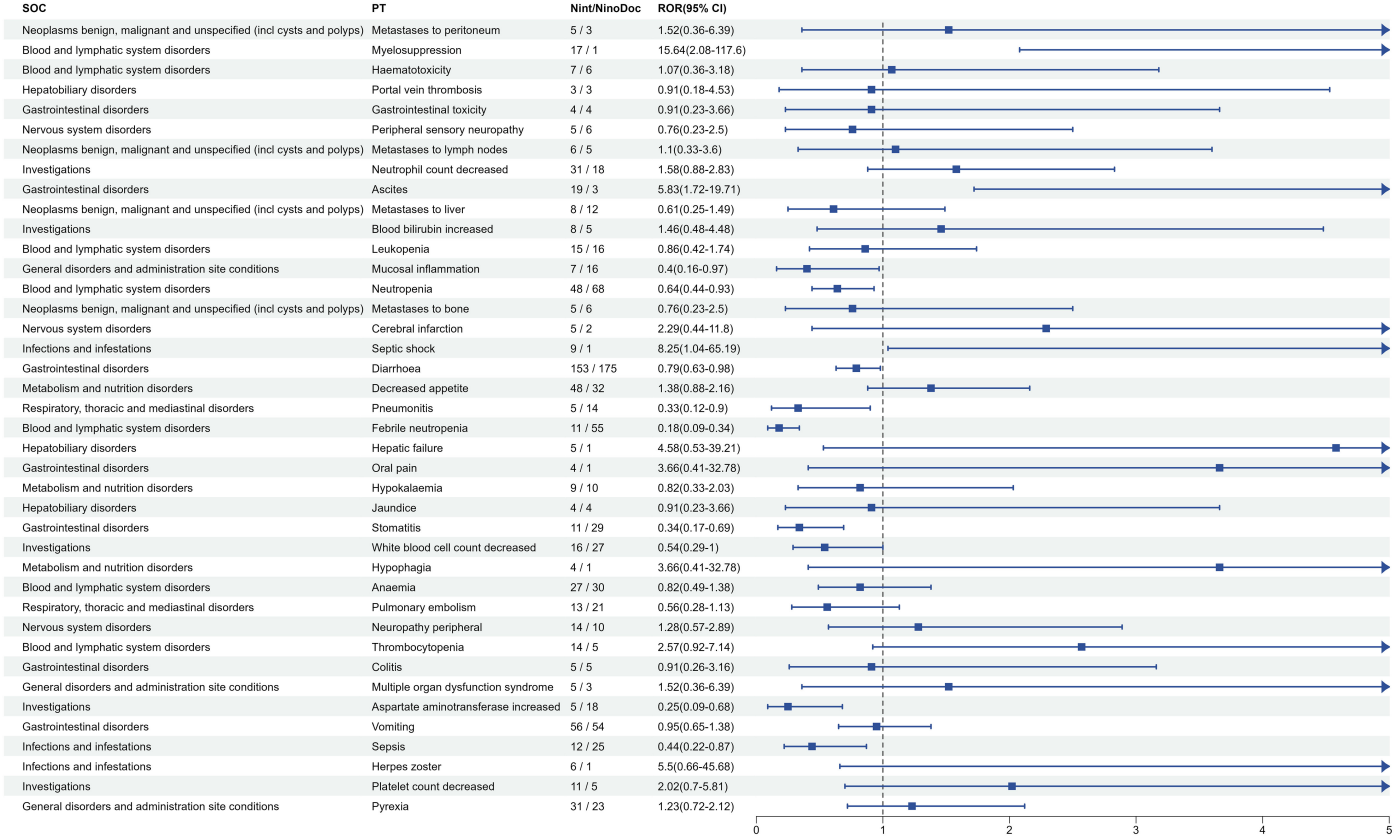
Comparison of AE signals between nintedanib monotherapy and docetaxel-nintedanib combination therapy at the PT level. SOC, system organ class; PT, Preferred term; Nint, nintedanib; NinoDoc, docetaxel-nintedanib combination therapy; ROR, reporting odds ratio; 95%CI, 95% confidence interval.

As **Figure 3** shown that the combination regimen was associated with a higher reporting frequency of hematologic toxicities, including febrile neutropenia, neutropenia, decreased white blood cell count and stomatitis. In contrast, several ADRs were reported with nintedanib monotherapy, including hepatic failure, cerebral infarction, thromboembolic events and blood bilirubin increased.

### 3.4 Stratification analysis

To explore whether demographic characteristics affect the pattern of ADR signals, we performed subgroup analyses at the PT level for both regimens using the OR method, stratified by age (< 65 vs ≥ 65 years) and sex.

In the combination therapy group, the safety profile of age on ADR was also significant. Elderly patients (age ≥ 65 years) showed elevated risks of severe infectious, respiratory and metabolic adverse events including pyrexia, pneumonia, pleural effusion, sepsis, respiratory failure, and agranulocytosis (**Supplementary Figure 1**). In contrast, the younger group (age < 65 years) was more likely to experience hair disorder, alopecia, depression, and anxiety. In the sex comparison (**Supplementary Figure 2**), female patients were prone to more types of ADR than male patients, the most likely being increased alanine aminotransferase, increased aspartate aminotransferase, hypokalaemia, haemoptysis, and increased gamma-glutamyl transferase. In contrast, male patients were more likely to experience adverse reactions such as neutropenia, dyspnea, asthenia, sepsis.

For docetaxel monotherapy, as shown in **Supplementary Figure 3**, the elderly group (age ≥ 65 years) showed a higher risk of most ADRs, especially in diarrhea, pyrexia, stomatitis, pneumonia, decreased appetite, respiratory failure, and agranulocytosis, with the most significant differences. At the sex level (**Supplementary Figure 4**), males had a higher risk of most severe ADR than females, including infections, hematopoietic suppression, and pulmonary complications. In contrast, females were more likely to report emotional and skin-related events such as alopecia, hair texture abnormal, depression, anxiety, acquired dacryostenosis, and increased aspartate aminotransferase.

After excluding lawyer-submitted reports, 39 of the 55 PT-level signals identified in the primary analysis remained detectable (**Supplementary Table 9**). Spearman’s rank correlation analysis demonstrated a significant positive correlation between ROR estimates derived from the original dataset and those obtained after exclusion of lawyer reports (ρ = 0.771, P < 0.001) (**Supplementary Figure 5**). Although several signals exhibited changes in ROR magnitude following exclusion of lawyer reports, the overall ranking pattern of the major safety signals remained broadly consistent. These findings suggest that reporting-source composition may influence the detectability and quantitative strength of certain signals, particularly those supported by relatively small numbers of reports, while the principal safety signal pattern remained generally stable.

### 3.5 Clinical priority scoring analysis

**Figure 4:**
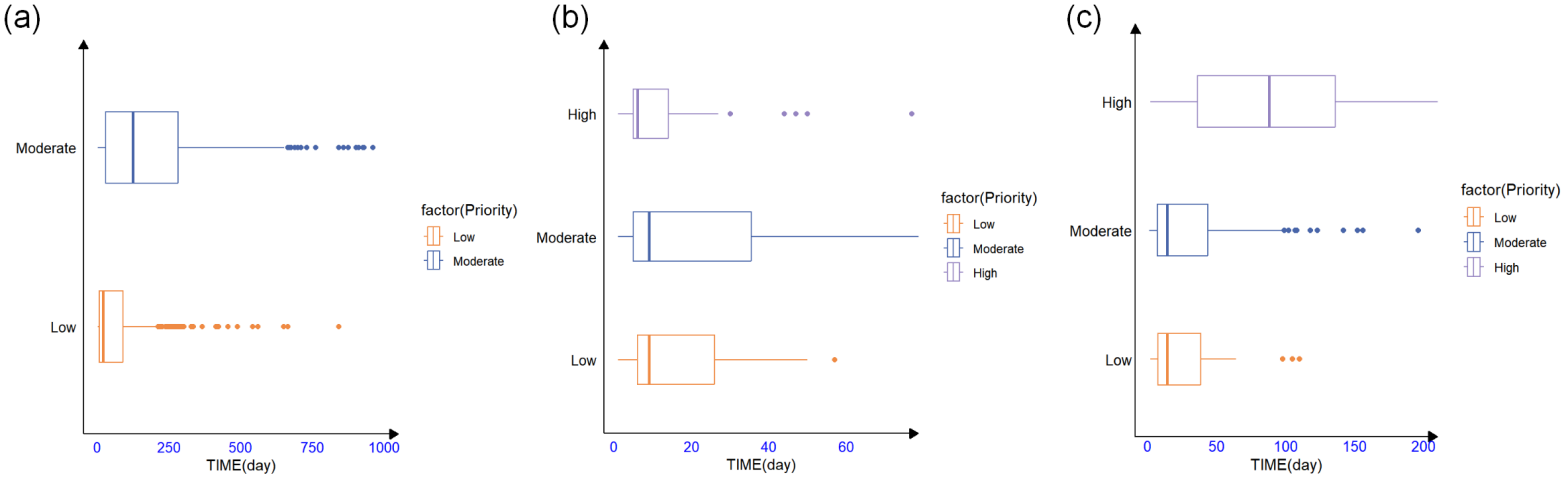
**(a)** Time-to-onset analysis of docetaxel monotherapy for different priority signals; **(b)** Time-to-onset analysis of docetaxel combined with nintedanib for different priority signals; **(c)** Time-to-onset analysis of nintedanib monotherapy for different priority signals.

Among the 54 signals for docetaxel monotherapy, 18 ADRs (31.5%) were classified as Important Medical Events (IMEs), while 3 (5.2%) met the criteria for Designated Medical Events (DMEs), including agranulocytosis, neutropenic sepsis, and neutropenic infection. Based on the clinical prioritization system, most ADRs (52.6%) were categorized as moderate priority, and the remaining ADRs (47.5%) were low clinical priority (**Supplementary Table 6**). The median time-to-onset was substantially longer for moderate-priority signals than for low-priority signals [124 (29–282) vs. 20 (7–89.5) days], as shown in **Figure 4a**.

Among the 78 signals in the combination therapy group, 27 ADRs (34.3%) were classified as IMEs, and 4 ADRs (5.1%) were classified as DMEs. The ADRs classified as DMEs were neutropenic sepsis, febrile neutropenia, intestinal perforation, and agranulocytosis. In addition, 4, 55, and 19 ADRs were identified as high, moderate, and low clinical priority, respectively. Among them, three AEs—febrile neutropenia, intestinal perforation, and sepsis—had the highest total clinical priority score of 6 points, being classified as high clinical priority events (**Supplementary Table 7**). The time-to-onset analysis of the combination therapy for different priority signals as shown in **Figure 4b**.

Among the 66 signals in the nintedanib monotherapy, a total of 32 AEs (48.4%) were classified as IMEs, and 3 ADRs (4.5%) were classified as DMEs. The ADRs classified as DMEs were febrile neutropenia, hepatic failure and pancreatitis. Among the identified signals, most ADRs (74.2%) had moderate clinical priority (**Supplementary Table 8**). The median time-to-onset was 124 (29–282) days for moderate-priority signals and 20 (7-89.5) days for low-priority signals, as shown in **Figure 4c**.

### 3.6 Cumulative incidence and time-to-onset analysis of ADR

**Figure 5:**
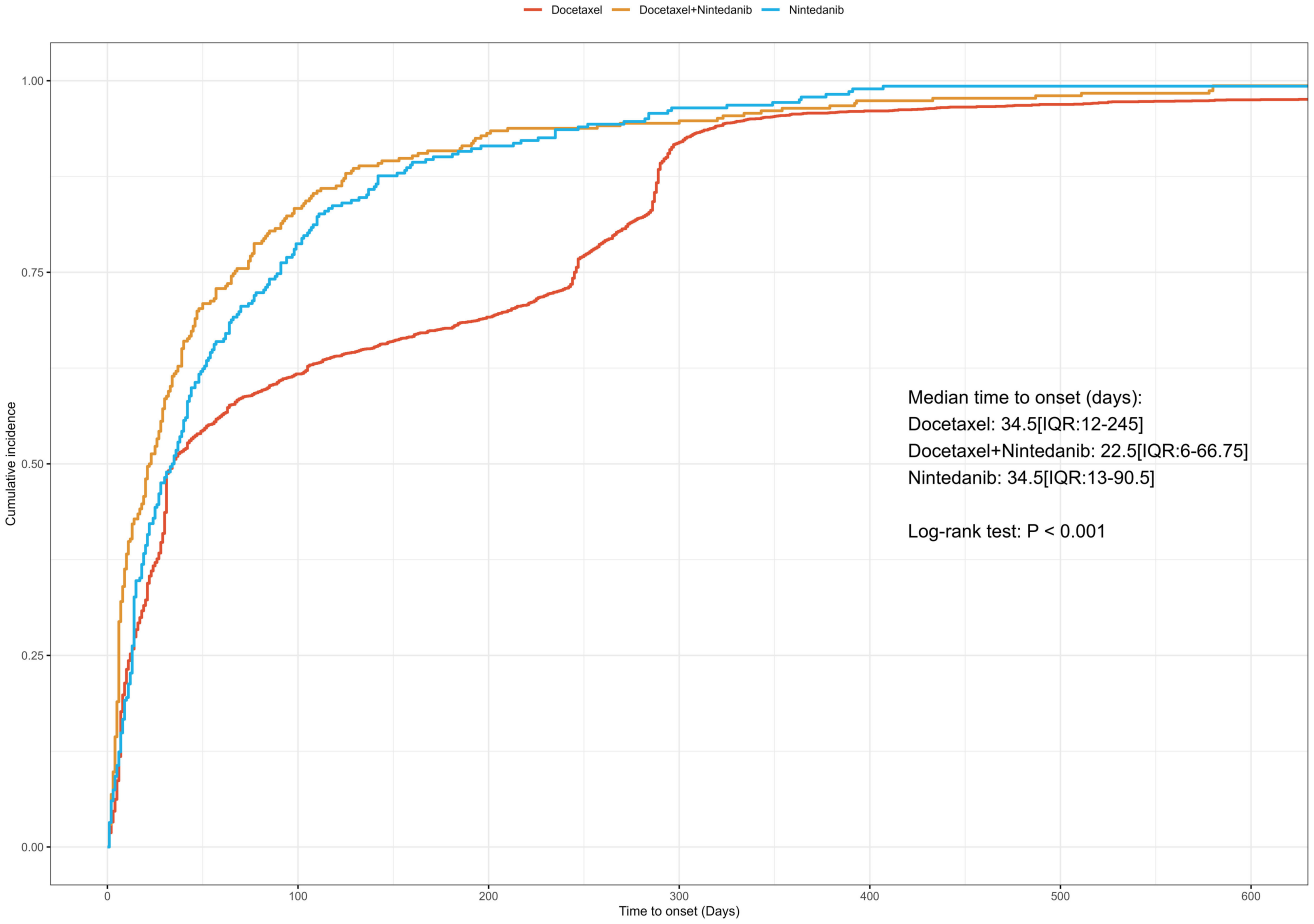
Cumulative distribution of time to adverse drug reactions for docetaxel, nintedanib and their combination.

TTO analysis revealed median ADR onset times of 34.50 (12.00–245.25) days for docetaxel, 34.50 (13.00–90.50) days for nintedanib, and an earlier 22.50 (6.00–67.25) days for combination therapy (**Table 3**). Most ADRs arose within 30 days post-administration: 43.66% for docetaxel, 47.30% for nintedanib, and 58.50% for combination therapy. In addition, WSP analysis showed that there was a statistically significant difference in the cumulative incidence of ADRs between patients receiving the three treatment regimens (**Figure 5**). The shape parameter β values of three regimens were < 1, indicating that most ADRs associated with the three regimens occurred in the early stage of drug administration and decreased over time, presenting an early failure curve. Furthermore, to explore the temporal patterns of ADRs for combination therapy stratified by individual characteristics, we conducted WSP analyses stratified by sex and age subgroups (**Supplementary Figure 6**). Log-rank tests revealed comparable cumulative ADR incidence curves across all sex and age subgroups receiving combination therapy, with no statistically significant intergroup differences in ADR onset trends.

**Table 3:** Weibull distribution of adverse drug reactions for docetaxel, nintedanib and their combination.

Database	Case (n)	Median(IQR)	Scale parameter α (95% CI)	Shape parameter β (95% CI)	Failure type
Docetaxel	8341	34.5 (12.00-245.25)	103.79 (98.00,109.58)	0.66 (0.64,0.68)	Early failure
nintedanib	937	34.5 (13-90.5)	60.27 (50.70, 69.84)	0.78 (0.71, 0.85)	Early failure
Nino-Doc	1459	22.5 (6.00-66.75)	46.27 (38.00,54.53)	0.67 (0.61,0.72)	Early failure

*When the shape parameter β <1, the 95%CI < 1, the incidence of ADR is considered to decline over time (early failure type).

Nino-Doc, docetaxel-nintedanib combination therapy.

## 4 Discussion

Based on real-world data from the FAERS database between 2014 and Q1 2025, this study systematically evaluated the ADR characteristics of docetaxel, nintedanib and their combination. Focused on identifying differences in risk profiles between the three treatment regimens, similarities and differences in ADR/E occurrence among patients with different characteristics, and the temporal patterns of ADR induction, this study comprehensively revealed the safety similarities and differences between three regimens, providing important references for rational clinical drug use.

At the SOC level for docetaxel monotherapy, the most frequently reported SOCs were skin and subcutaneous tissue disorders, general disorders and administration site conditions, and psychiatric disorders, among which the former two generated positive signals. As a taxane chemotherapeutic drug, docetaxel exerts cytotoxicity on rapidly dividing cells, commonly causing typical cutaneous ADRs such as alopecia and hair abnormalities. This observation matches the established safety profile of single-agent docetaxel reported in previous clinical researches (33). For nintedanib monotherapy, its frequently reported SOCs included gastrointestinal disorders and general disorders and administration site conditions, with significant disproportionality signals detected in gastrointestinal disorders, blood and lymphatic system disorders. Further cross-group comparison confirmed that metabolic and electrolyte disturbances, musculoskeletal pain and asthenia were the hallmark adverse events triggered by nintedanib alone. The nintedanib blocks the VEGFR pathway to inhibit gastrointestinal mucosal angiogenesis, which reduces mucosal blood supply and destroys intestinal barrier function, thereby inducing digestive tract adverse reactions (34). In combination treatment, the most common SOCs were gastrointestinal disorders, general disorders and administration site conditions, and benign, malignant and unspecified neoplasms, and all three categories met the dual signal positive criteria (lower limit of ROR 95% CI > 1 and IC025 > 0). As illustrated in **Figure 1**, combination therapy significantly elevated the ADR risks of multiple organ systems. Further disproportionality analysis at the SOC level showed that combination therapy significantly increased the risk of hematological and lymphatic system disorders. A phase I study by Okamoto et al. also showed that the incidence of drug-related neutropenia was as high as 95% in 42 Japanese NSCLC patients (35). Reck M et al. also reported neutropenia as a common adverse event (36). Due to docetaxel’s inherent myelosuppressive properties, neutropenia and its complications are expected toxicities of this combination regimen, suggesting that enhanced monitoring and management of related systems are necessary during combination treatment.

In signal detection at the PT level, the combination therapy exhibited a broad spectrum of significant ADR signals, the comparative analyses indicated that the toxicity profile was characterized by relatively higher reporting frequencies of hematologic, gastrointestinal, infectious, thromboembolic, and hepatic events. In contrast, docetaxel monotherapy was mainly associated with skin appendage disorders and psychosocial-related ADRs, whereas nintedanib monotherapy showed relatively greater reporting of hepatic and vascular toxicities. The enrichment of infection-related events for the combination therapy, including mucosal infection, pneumonia, and sepsis, may reflect the combined effects of chemotherapy-induced myelosuppression and antiangiogenic therapy–associated impairment of mucosal repair. Bone marrow suppression reduces host immune defense, whereas inhibition of VEGF-, PDGFR-, and FGFR-mediated signaling may compromise vascular integrity and tissue regeneration (37). Notably, portal vein thrombosis was a rare but serious vascular event, which was a special form of anti-angiogenic drug-related thromboembolic events. The safety analysis of the LUME-Lung 1 study reported an overall incidence of thromboembolic events (5.1%). Meta-analyses have shown that anti-angiogenic drugs are associated with an increased risk of arterial and venous thromboembolism (38, 39). FAERS signals also suggest that nintedanib’s anti-angiogenic effect may increase the risk of thrombosis, and clinicians should be alert to warning symptoms such as abdominal pain and portal hypertension. In addition, docetaxel’s hepatic metabolism may share hepatotoxic pathways with nintedanib, leading to a significant increase in the reporting rate of liver function abnormalities. Our results are consistent with adverse reactions documented in multiple previous studies, further confirming the authenticity of our findings (14, 14, 40).

In the subgroup comparison analysis of ADRs among different ages and genders, for docetaxel monotherapy, elderly patients aged ≥ 65 years had a significantly higher risk of severe ADRs such as diarrhea, pyrexia, pneumonia, and respiratory failure than younger patients, which is closely related to the decline in liver and kidney function and bone marrow reserve capacity in elderly patients. Previous studies have mentioned that the risk of chemotherapy adverse reactions is usually higher in the elderly than in younger patients (41, 42). In contrast, younger patients were more prone to hair abnormalities and psychosocial reactions, which is associated with younger patients’ higher attention to appearance and quality of life and lower psychological stress threshold. At the gender level, female patients in the monotherapy group had a much higher risk of alopecia, depression, and anxiety than male patients, while male patients were more likely to experience gastrointestinal and hematological system events. This difference is related to the regulatory effect of sex hormones on drug-metabolizing enzymes (such as CYP3A4). For the combination therapy group, it was found that elderly patients were more likely to experience ADRs such as pyrexia, pneumonia, and pleural effusion, while younger patients were mainly affected by hair abnormalities and psychiatric symptoms; in female patients, the risk of liver function-related index abnormalities (such as increased alanine aminotransferase) was higher, which is the most common grade ≥ 3 adverse event of nintedanib combined with docetaxel. In contrast, male patients were more likely to experience neutropenia, dyspnea, sepsis, and other adverse reactions. These subgroup differences suggest that individualized risk management is required in clinical treatment: for elderly patients, drug doses should be appropriately reduced or supportive treatment strengthened; for female patients, liver function should be monitored; for male patients, focus should be placed on preventing infections and myelosuppression.

We used clinical priority scoring to further identify high-risk events of the three treatment regimens. In contrast to monotherapy, the combination therapy had more DMEs, and the onset time of high-priority signals was earlier (6 days vs 124 days). High-priority AEs in combination therapy were more concentrated in hematology, gastrointestinal tract, and infections, while monotherapy was biased toward psychiatric or hair-related adverse reactions. Febrile neutropenia, intestinal perforation, and sepsis in the combination therapy regimen had a clinical priority score of 6 points, being high-priority events. Neutropenic sepsis is usually the most common fatal adverse reaction of chemotherapy, while intestinal perforation, as a rare but serious adverse reaction of nintedanib, requires immediate drug withdrawal and intervention, which is highly consistent with previous literature reports (43). At the same time, we analyzed the median onset time of clinically prioritized signals. The TTO differences among signals of different clinical priorities suggest that high-risk adverse reactions of combination therapy mostly occur in the early stage of treatment, requiring enhanced monitoring in the early stage of drug use (especially the first 1–2 weeks), while moderate to severe adverse reactions of monotherapy may occur delayed, requiring long-term follow-up.

Through WSP analysis, we found that the temporal pattern of ADRs in the three treatment regimens showed an early failure type (β < 1), and most ADRs were concentrated in the early stage of drug use. The median onset time of combination therapy (22.50 days) was earlier than that of monotherapy (34.50 days), indicating that ADRs of combination therapy occur earlier, which may be related to the early mucosal damage and myelosuppression caused by nintedanib’s anti-angiogenic mechanism, suggesting that a more intensive monitoring plan should be formulated in the early stage of combination therapy. In the temporal analysis of each subgroup, there was no significant difference in the cumulative incidence of ADRs among different genders and age groups in the combination therapy regimen, which may be related to the higher tumor burden of patients receiving combination therapy, while the subgroup differences in the monotherapy group reflect the possible impact of basic health status on chemotherapy toxicity.

Several limitations inherent to spontaneous reporting systems must be acknowledged. First, the inherent biases of FAERS (e.g., under-reporting, over-reporting of serious events, reporting trends) cannot be eliminated (44–46). To avoid interference from false-positive signals, we performed Bonferroni correction on the signals obtained by the ROR method and used the Bayesian-based Information Component (IC) method for multiple signal identification. Second, since disproportionality analysis can only indicate a potential association between a drug and an adverse event, the association results may be affected by confounding factors and cannot confirm whether the association is causal (27). Therefore, when applying this method, we conducted subgroup analyses at the demographic characteristic level to ensure the accuracy of the results. In addition, we excluded all irrelevant events, including product issues, medication errors (administration, confusion, monitoring, etc.), and social circumstances. Lastly, although FDA-recommended deduplication procedures were applied, residual non-independence among reports cannot be completely excluded because FAERS lacks unique patient and institution identifiers.

Reporter-source heterogeneity is a recognized limitation of spontaneous reporting systems such as FAERS (47, 48). In the present study, a substantial proportion of reports in the monotherapy group originated from lawyers, raising concerns regarding potential reporting bias. Sensitivity analyses demonstrated that exclusion of lawyer-submitted reports resulted in the loss of a subset of detected signals, indicating that reporting source may influence signal detectability. However, a significant correlation in ROR estimates was observed before and after exclusion, and the relative ranking of the major safety signals remained largely unchanged. These findings suggest that lawyer-derived reports may contribute to the identification of certain weaker or less frequently reported signals, whereas the overall pattern of the principal safety findings was relatively robust. Nevertheless, the potential influence of reporting-source imbalance should be considered when interpreting disproportionality results derived from spontaneous reporting databases.

This study systematically analyzed the combination-associated safety profile and clinical priority of docetaxel combined with nintedanib dual-pathway inhibition in the treatment of advanced NSCLC through real-world data. In addition to signal detection methods, we also combined clinical priority scoring and WSP temporal trend analysis, which not only clarified toxicity differences but also quantified risk levels and onset times, providing multi-dimensional evidence for clinical risk management. Although the combination therapy regimen showed certain ADR risks, multiple studies have shown that the safety of nintedanib combined with docetaxel, pemetrexed, etc., is controllable and can be managed through appropriate treatment, dose interruption, or dose adjustment. The LUME-Lung 1 study also confirmed that this combination therapy can significantly prolong the overall survival of specific NSCLC populations (especially adenocarcinoma patients), so it must be weighed against its significant survival benefits. Moreover, the purpose of our study is not to question the effectiveness of this regimen, but to provide evidence-based basis for optimizing its clinical management. Despite certain limitations, the results of this study still have important practical guiding value and are expected to improve the treatment safety and quality of life of patients with advanced NSCLC.

## Data Availability

The original contributions presented in the study are included in the article/**Supplementary Material**. Further inquiries can be directed to the corresponding author.
