# Supplementary material for: Synergistic toxicity and clinical prioritization of docetaxel combined with nintedanib for non-small cell lung cancer: a real-world safety analysis using the FAERS database
Source: Front Oncol. 2026 Jul 27;16:1836198. doi: 10.3389/fonc.2026.1836198 (PMC13453697; doi:10.3389/fonc.2026.1836198)
Supplement: Supplementary file 1 [file DataSheet1.docx]

# *Supplementary Material*

**Supplementary Table 1** The detailed formulas for disproportionality analysis.

| **Algorithms** | **Equation** | **Threshold** |
| --- | --- | --- |
| ROR | $ROR=\frac{a/c}{b/d}$ | lower limit of 95% CI > l, a≥ 3 |
|  | $95\%CI=e^{ln(ROR)\pm1.96\sqrt{(\frac{1}{a}+\frac{1}{b}+\frac{1}{c}+\frac{1}{d})}}$ |  |
| BCPNN | $IC=={log}_{2}\frac{a(a+b+c+d)}{(a+b)(a+c)}$ | IC025>0 |
|  | ${IC025=log}_{2}\frac{a+0.5}{E+0.5}-{3.3(a+0.5)}^{-1/2}-{2(a+0.5)}^{-3/2}$ |  |
| MHRA | PRR=a(c+d)/c/(a+b) | PRR≥2, χ^2^≥4, a≥3 |
|  | χ^2^=[(ad-bc)^2](a+b+c+d)/[(a+b)(c+d)(a+c)(b+d)] |  |

Notes: ROR, reporting odds ratio; 95%CI, 95% confidence interval; IC, information component; BCPNN, bayesian confidence propagation neural network; χ^2^, chi-squared; a, the number of reports;

Equation: a, the number of target adverse events reports of the target drug(Patients receiving docetaxel with or without nintedanib); b, The number of other adverse events reports of the target drug; c, the number of target adverse events reports of non-target drugs; d, the number of other adverse events reports of non-target drugs; E:(a+b)(a+c)/(a+b+c+d).

**Supplementary Table 2**. A rating scale assessing clinical priority of disproportionality signals.

| **Assessment items** | **0 point** | **1 point** | **2 points** |
| --- | --- | --- | --- |
| Reporting rate | 0-1% | 1-10% | >10% |
| Signal stability | 1 of 3 | 2 of 3 | 3 of 3 |
| Mortality proportion | <25% | 25-50% | >50% |
| Clinical relevance (IMEs/ DMEs) | - | IME | DME |

Signal stability: consistency across disproportionality analyses.

Mortality Proportion: The ratio of fatal outcomes to total reported cases for a specific adverse event (AE), expressed as a percentage.

Clinical relevance: Important Medical Events (IMEs) and Designated Medical Events (DMEs) are categories defined and regularly revised by the European Medicines Agency (EMA).

**Supplementary Table 3** Signal strength for docetaxel associated reports at the SOC level

| SOC | Cases(n) | ROR | 95% CI | | IC | IC_025_ |
| --- | --- | --- | --- | --- | --- | --- |
|  |  |  | Low bound | Up bound |  |  |
| Skin And Subcutaneous Tissue Disorders** | 23150 | 29.10 | 28.41 | 29.81 | 2.87 | 2.84 |
| General Disorders And Administration Site Conditions | 1905 | 0.30 | 0.28 | 0.31 | -1.53 | -1.59 |
| Psychiatric Disorders** | 1709 | 2.65 | 2.52 | 2.79 | 1.26 | 1.18 |
| Gastrointestinal Disorders | 1682 | 0.31 | 0.29 | 0.32 | -1.51 | -1.58 |
| Respiratory, Thoracic And Mediastinal Disorders | 901 | 0.25 | 0.24 | 0.27 | -1.82 | -1.92 |
| Investigations | 868 | 0.33 | 0.30 | 0.35 | -1.50 | -1.60 |
| Blood And Lymphatic System Disorders | 845 | 0.59 | 0.55 | 0.64 | -0.70 | -0.80 |
| Infections And Infestations | 827 | 0.40 | 0.37 | 0.42 | -1.24 | -1.34 |
| Neoplasms Benign, Malignant And Unspecified (Incl Cysts And Polyps) | 732 | 0.35 | 0.32 | 0.37 | -1.43 | -1.53 |
| Nervous System Disorders | 538 | 0.23 | 0.22 | 0.26 | -1.97 | -2.09 |
| Metabolism And Nutrition Disorders | 345 | 0.27 | 0.24 | 0.30 | -1.82 | -1.97 |
| Eye Disorders | 330 | 0.77 | 0.69 | 0.86 | -0.36 | -0.52 |
| Injury, Poisoning And Procedural Complications | 317 | 0.18 | 0.16 | 0.20 | -2.38 | -2.54 |
| Musculoskeletal And Connective Tissue Disorders | 288 | 0.26 | 0.23 | 0.29 | -1.87 | -2.04 |
| Vascular Disorders | 251 | 0.36 | 0.32 | 0.41 | -1.41 | -1.59 |
| Cardiac Disorders | 242 | 0.27 | 0.24 | 0.31 | -1.80 | -1.98 |
| Renal And Urinary Disorders | 175 | 0.23 | 0.20 | 0.27 | -2.04 | -2.25 |
| Hepatobiliary Disorders | 133 | 0.19 | 0.16 | 0.23 | -2.29 | -2.53 |
| Immune System Disorders | 65 | 0.23 | 0.18 | 0.29 | -2.07 | -2.41 |
| Endocrine Disorders | 51 | 0.18 | 0.14 | 0.24 | -2.39 | -2.77 |
| Ear And Labyrinth Disorders | 32 | 0.28 | 0.20 | 0.40 | -1.76 | -2.24 |
| Congenital, Familial And Genetic Disorders | 19 | 0.25 | 0.16 | 0.39 | -1.95 | -2.54 |
| Reproductive System And Breast Disorders | 15 | 0.23 | 0.14 | 0.38 | -2.06 | -2.71 |

Notes: Asterisks (*) indicate that the signal is statistically significant in all two formulas.

Abbreviations: SOC, system organ class; RORL, ROR 95% confidence interval lower limit; IC, information component; IC_025_: 95% confidence interval of IC.

**Supplementary Table 4** Signal strength for docetaxel-nintedanibin combination at the system organ class level

| SOC | Cases(n) | ROR | 95% CI | | IC | IC_025_ |
| --- | --- | --- | --- | --- | --- | --- |
|  |  |  | Low bound | Up bound |  |  |
| Gastrointestinal Disorders* | 447 | 2.65 | 2.39 | 2.93 | 1.22 | 1.06 |
| General Disorders And Administration Site Conditions | 350 | 0.82 | 0.73 | 0.92 | -0.24 | -0.4 |
| Neoplasms Benign, Malignant And Unspecified (Incl Cysts And Polyps)* | 301 | 5.16 | 4.57 | 5.83 | 2.21 | 2.01 |
| Blood And Lymphatic System Disorders* | 209 | 5.99 | 5.19 | 6.9 | 2.47 | 2.23 |
| Investigations* | 174 | 1.33 | 1.14 | 1.55 | 0.38 | 0.15 |
| Respiratory, Thoracic And Mediastinal Disorders* | 174 | 1.68 | 1.44 | 1.96 | 0.71 | 0.47 |
| Infections And Infestations | 144 | 1.16 | 0.98 | 1.38 | 0.2 | -0.04 |
| Nervous System Disorders | 109 | 0.58 | 0.48 | 0.7 | -0.73 | -1.01 |
| Skin And Subcutaneous Tissue Disorders | 91 | 0.69 | 0.56 | 0.85 | -0.51 | -0.81 |
| Metabolism And Nutrition Disorders* | 73 | 1.55 | 1.23 | 1.96 | 0.62 | 0.26 |
| Vascular Disorders | 41 | 0.9 | 0.66 | 1.23 | -0.15 | -0.59 |
| Musculoskeletal And Connective Tissue Disorders | 39 | 0.31 | 0.23 | 0.43 | -1.62 | -2.05 |
| Hepatobiliary Disorders* | 34 | 1.78 | 1.27 | 2.5 | 0.83 | 0.3 |
| Renal And Urinary Disorders | 30 | 0.69 | 0.48 | 0.98 | -0.54 | -1.04 |
| Cardiac Disorders | 26 | 0.51 | 0.34 | 0.75 | -0.97 | -1.5 |
| Injury, Poisoning And Procedural Complications | 23 | 0.08 | 0.05 | 0.11 | -3.55 | -4.09 |
| Psychiatric Disorders | 9 | 0.07 | 0.04 | 0.13 | -3.76 | -4.54 |
| Eye Disorders | 7 | 0.15 | 0.07 | 0.32 | -2.7 | -3.56 |
| Ear And Labyrinth Disorders | 7 | 0.7 | 0.33 | 1.46 | -0.52 | -1.48 |
| Endocrine Disorders | 6 | 1 | 0.45 | 2.22 | 0 | -1.1 |
| Immune System Disorders | 6 | 0.22 | 0.1 | 0.48 | -2.19 | -3.11 |
| Congenital, Familial And Genetic Disorders | 3 | 0.48 | 0.16 | 1.49 | -1.05 | -2.3 |
| Reproductive System And Breast Disorders | 2 | 0.11 | 0.03 | 0.43 | -3.2 | -4.36 |

Asterisks (*) indicate that the signal is statistically significant in all two formulas.

Abbreviations: SOC, system organ class; RORL, ROR 95% confidence interval lower limit; IC, information component; IC_025_: 95% confidence interval of IC.

**Supplementary Table 5** Signal strength for nintedanib at the system organ class level

| SOC | Cases(n) | ROR | 95% CI | | IC | IC_025_ |
| --- | --- | --- | --- | --- | --- | --- |
|  |  |  | Low bound | Up bound |  |  |
| Gastrointestinal disorders* | 518 | 2.83 | 2.57 | 3.12 | 1.3 | 1.15 |
| General disorders and administration site conditions | 466 | 1.02 | 0.92 | 1.13 | 0.03 | -0.12 |
| Injury, poisoning and procedural complications | 226 | 0.77 | 0.67 | 0.88 | -0.34 | -0.54 |
| Neoplasms benign, malignant and unspecified (incl cysts and polyps)* | 215 | 5.82 | 5.06 | 6.69 | 2.43 | 2.2 |
| Investigations | 182 | 1.16 | 1 | 1.35 | 0.2 | -0.02 |
| Blood and lymphatic system disorders* | 161 | 4.35 | 3.71 | 5.11 | 2.05 | 1.79 |
| Infections and infestations | 132 | 1.09 | 0.91 | 1.29 | 0.11 | -0.15 |
| Nervous system disorders | 108 | 0.51 | 0.42 | 0.61 | -0.92 | -1.2 |
| Metabolism and nutrition disorders* | 107 | 2 | 1.65 | 2.43 | 0.97 | 0.67 |
| Respiratory, thoracic and mediastinal disorders | 105 | 0.83 | 0.68 | 1 | -0.26 | -0.55 |
| Hepatobiliary disorders* | 55 | 2.6 | 1.99 | 3.4 | 1.36 | 0.93 |
| Skin and subcutaneous tissue disorders | 54 | 0.39 | 0.29 | 0.51 | -1.33 | -1.7 |
| Vascular disorders | 52 | 0.93 | 0.71 | 1.23 | -0.1 | -0.5 |
| Musculoskeletal and connective tissue disorders | 34 | 0.24 | 0.17 | 0.34 | -2 | -2.46 |
| Renal and urinary disorders | 26 | 0.55 | 0.37 | 0.81 | -0.86 | -1.39 |
| Cardiac disorders | 23 | 0.38 | 0.25 | 0.58 | -1.36 | -1.92 |
| Immune system disorders | 19 | 0.6 | 0.38 | 0.94 | -0.74 | -1.36 |
| Psychiatric disorders | 12 | 0.07 | 0.04 | 0.12 | -3.75 | -4.45 |
| Eye disorders | 7 | 0.15 | 0.07 | 0.31 | -2.75 | -3.61 |
| Congenital, familial and genetic disorders | 1 | 0.16 | 0.02 | 1.11 | -2.68 | -3.93 |
| Ear and labyrinth disorders | 1 | 0.09 | 0.01 | 0.64 | -3.46 | -4.62 |

Asterisks (*) indicate that the signal is statistically significant in all two formulas.

Abbreviations: SOC, system organ class; RORL, ROR 95% confidence interval lower limit; IC, information component; IC_025_: 95% confidence interval of IC.

**Supplementary Table 6** Signal strength of reports of docetaxel at the preferred term and the clinical priority assessing results

| Preferred term | Cases (n) | ROR (95%CI) | IC (IC_025_) | MHRA | Death (n) | DEM/ IME | Priority level (score) | BH-FDR | *Bonferroni*_*p* |
| --- | --- | --- | --- | --- | --- | --- | --- | --- | --- |
| Psychological trauma | 202 | 3976.4 (557.39-28367.70) | 4.36 (3.93) | 3953.80 (3933.42) | 0 | NA | Low(2) | p<0.001 | p<0.001 |
| Madarosis | 3966 | 1648.57 (1256.91-2162.28) | 4.34 (4.27) | 1464.67 (76922.18) | 21 | NA | Moderate(4) | p<0.001 | p<0.001 |
| Hair disorder | 4417 | 1451.74 (1142.23-1845.11) | 4.34 (4.27) | 1271.40 (85535.18) | 25 | NA | Moderate(4) | p<0.001 | p<0.001 |
| Hair colour changes | 4423 | 1051.79 (857.17-1290.59) | 4.33 (4.26) | 920.98 (85112.86) | 25 | NA | Moderate(4) | p<0.001 | p<0.001 |
| Hair texture abnormal | 4421 | 567.85 (487.82-661.00) | 4.31 (4.24) | 497.32 (83446.64) | 25 | NA | Moderate(4) | p<0.001 | p<0.001 |
| Self esteem decreased | 25 | 163.22 (49.28-540.64) | 4.20 (2.65) | 163.11 (431.58) | 0 | NA | Low(2) | p<0.001 | p<0.001 |
| Post-traumatic stress disorder | 42 | 91.45 (44.51-187.88) | 4.08 (3.01) | 91.34 (662.31) | 0 | NA | Low(2) | p<0.001 | p<0.001 |
| Dacryostenosis acquired | 94 | 87.84 (54.73-141.00) | 4.07 (3.44) | 87.61 (1470) | 0 | NA | Low(2) | p<0.001 | p<0.001 |
| Ecthyma | 4 | 78.30 (8.75-700.60) | 4.04 (0.21) | 78.29 (61.05) | 0 | NA | Low(2) | p<0.001 | p<0.001 |
| Pneumonia salmonella | 4 | 78.30 (8.75-700.60) | 4.04 (0.21) | 78.29 (61.05) | 1 | IME | Moderate(4) | p<0.001 | p<0.001 |
| Alopecia | 5360 | 61.20 (58.07-64.50) | 3.90 (3.85) | 52.12 (74117.9) | 40 | NA | Moderate(4) | p<0.001 | p<0.001 |
| Hair injury | 9 | 58.73 (15.90-216.96) | 3.95 (1.40) | 58.72 (127.66) | 0 | NA | Low(2) | p<0.001 | p<0.001 |
| Deformity | 6 | 39.15 (9.79-156.56) | 3.78 (0.81) | 39.15 (74.35) | 0 | NA | Low(2) | p<0.001 | p<0.001 |
| Emotional distress | 132 | 31.62 (24.00-41.66) | 3.67 (3.22) | 31.51 (1494.55) | 0 | NA | Low(2) | p<0.001 | p<0.001 |
| Ischaemic enteritis | 4 | 26.10 (5.84-116.63) | 3.56 (0.20) | 26.10 (41.38) | 0 | IME | Moderate(3) | p<0.001 | p<0.001 |
| Orthostatic intolerance | 4 | 26.10 (5.84-116.63) | 3.56 (0.20) | 26.10 (41.38) | 0 | NA | Low(2) | p<0.001 | p<0.001 |
| Osmotic demyelination syndrome | 4 | 26.10 (5.84-116.63) | 3.56 (0.20) | 26.10 (41.38) | 0 | IME | Moderate(3) | p<0.001 | p<0.001 |
| Hypereosinophilic syndrome | 11 | 21.54 (9.15-50.71) | 3.43 (1.50) | 21.53 (102.55) | 11 | IME | Moderate(5) | p<0.001 | p<0.001 |
| Mucosal infection | 12 | 19.58 (8.80-43.59) | 3.36 (1.57) | 19.57 (105.75) | 1 | NA | Low(2) | p<0.001 | p<0.001 |
| Salmonella sepsis | 4 | 19.58 (4.90-78.28) | 3.36 (0.19) | 19.57 (35.25) | 1 | IME | Moderate(4) | p<0.001 | p<0.001 |
| Splenic vein thrombosis | 4 | 11.19 (3.27-38.21) | 2.9 (0.12) | 11.18 (23.61) | 4 | IME | Moderate(5) | p<0.001 | 0.002 |
| Bipolar disorder | 7 | 10.54 (4.21-26.42) | 2.85 (0.79) | 10.54 (39.29) | 0 | IME | Moderate(3) | p<0.001 | p<0.001 |
| Lacrimation increased | 134 | 9.33 (7.60-11.47) | 2.73 (2.38) | 9.30 (673.33) | 0 | NA | Low(2) | p<0.001 | p<0.001 |
| Major depression | 10 | 9.32 (4.39-19.80) | 2.73 (1.10) | 9.32 (50.32) | 0 | IME | Moderate(3) | p<0.001 | p<0.001 |
| Mediastinitis | 8 | 9.21 (3.98-21.35) | 2.72 (0.88) | 9.21 (39.82) | 0 | IME | Moderate(3) | p<0.001 | p<0.001 |
| Depression | 622 | 9.07 (8.25-9.98) | 2.69 (2.54) | 8.93 (3016.72) | 1 | NA | Moderate(3) | p<0.001 | p<0.001 |
| Necrotising fasciitis | 10 | 8.90 (4.21-18.79) | 2.68 (1.07) | 8.90 (48.19) | 0 | IME | Moderate(3) | p<0.001 | p<0.001 |
| Coating in mouth | 4 | 8.70 (2.68-28.25) | 2.66 (0.07) | 8.70 (18.87) | 4 | NA | Moderate(4) | p<0.001 | 0.012 |
| Eye injury | 7 | 8.56 (3.52-20.82) | 2.65 (0.71) | 8.56 (32.53) | 0 | NA | Low(2) | p<0.001 | p<0.001 |
| Obstruction gastric | 9 | 8.01 (3.69-17.39) | 2.58 (0.92) | 8.01 (39.17) | 0 | IME | Moderate(3) | p<0.001 | p<0.001 |
| Pathogen resistance | 11 | 7.69 (3.83-15.45) | 2.54 (1.08) | 7.69 (45.96) | 6 | NA | Moderate(4) | p<0.001 | p<0.001 |
| Anxiety | 465 | 7.38 (6.63-8.21) | 2.48 (2.32) | 7.29 (1843.88) | 2 | NA | Moderate(3) | p<0.001 | p<0.001 |
| Hypoventilation | 6 | 6.18 (2.47-15.48) | 2.30 (0.40) | 6.18 (19.80) | 0 | IME | Moderate(3) | p<0.001 | 0.004 |
| Haemolytic uraemic syndrome | 11 | 5.67 (2.90-11.09) | 2.21 (0.88) | 5.67 (32.78) | 0 | IME | Moderate(3) | p<0.001 | p<0.001 |
| Epidermolysis | 6 | 5.59 (2.26-13.86) | 2.19 (0.35) | 5.59 (17.60) | 6 | IME | Moderate(5) | p<0.001 | 0.010 |
| Neutropenic infection | 6 | 5.34 (2.16-13.17) | 2.14 (0.32) | 5.34 (16.62) | 3 | DEM | Moderate(5) | p<0.001 | 0.015 |
| Onycholysis | 9 | 5.18 (2.49-10.81) | 2.11 (0.66) | 5.18 (24.01) | 0 | NA | Low(2) | p<0.001 | p<0.001 |
| Atypical haemolytic uraemic syndrome | 5 | 5.15 (1.92-13.80) | 2.10 (0.13) | 5.15 (13.24) | 0 | IME | Moderate(3) | 0.002 | 0.084 |
| Oesophageal fistula | 6 | 4.89 (2.00-11.97) | 2.04 (0.27) | 4.89 (14.87) | 0 | IME | Moderate(3) | p<0.001 | 0.032 |
| Personality change | 7 | 4.57 (2.01-10.40) | 1.96 (0.37) | 4.57 (15.81) | 0 | NA | Low(2) | p<0.001 | 0.018 |
| Blepharitis | 8 | 4.02 (1.88-8.59) | 1.81 (0.38) | 4.02 (15.03) | 0 | NA | Low(2) | p<0.001 | 0.022 |
| Traumatic lung injury | 10 | 3.06 (1.57-5.96) | 1.48 (0.31) | 3.06 (11.98) | 0 | NA | Low(2) | 0.002 | 0.077 |
| Emotional disorder | 9 | 2.75 (1.37-5.53) | 1.34 (0.15) | 2.75 (8.80) | 0 | NA | Low(2) | 0.007 | 0.380 |
| Venous thrombosis | 13 | 2.55 (1.43-4.54) | 1.24 (0.28) | 2.54 (10.79) | 3 | NA | Low(2) | 0.002 | 0.117 |
| Mental disorder | 23 | 2.47 (1.60-3.82) | 1.21 (0.5) | 2.47 (17.93) | 0 | NA | Low(2) | p<0.001 | 0.003 |
| Onychomadesis | 12 | 2.16 (1.19-3.91) | 1.03 (0.07) | 2.15 (6.69) | 1 | NA | Low(2) | 0.018 | 0.947 |
| Stress | 26 | 1.91 (1.28-2.86) | 0.87 (0.24) | 1.91 (10.33) | 0 | NA | Low(1) | 0.002 | 0.113 |
| Polyneuropathy | 34 | 1.90 (1.34-2.71) | 0.87 (0.32) | 1.90 (13.25) | 24 | IME | Moderate(4) | p<0.001 | 0.023 |
| Agranulocytosis | 43 | 1.82 (1.33-2.49) | 0.81 (0.33) | 1.82 (14.59) | 2 | DEM | Moderate(3) | p<0.001 | 0.011 |
| Peripheral sensory neuropathy | 26 | 1.81 (1.21-2.71) | 0.80 (0.18) | 1.81 (8.65) | 3 | NA | Low(1) | 0.005 | 0.269 |
| Therapeutic response decreased | 24 | 1.79 (1.18-2.71) | 0.78 (0.14) | 1.79 (7.61) | 7 | NA | Low(2) | 0.009 | 0.472 |
| Neutropenic sepsis | 37 | 1.76 (1.26-2.46) | 0.76 (0.24) | 1.76 (11.10) | 10 | DEM | Moderate(4) | 0.001 | 0.069 |
| White blood cell count decreased | 168 | 1.43 (1.22-1.67) | 0.48 (0.25) | 1.43 (20.04) | 32 | NA | Low(1) | p<0.001 | p<0.001 |
| Stomatitis | 140 | 1.35 (1.14-1.61) | 0.41 (0.15) | 1.35 (11.95) | 31 | NA | Low(1) | p<0.001 | 0.035 |

Abbreviations: SOC, system organ class; RORL, ROR 95% confidence interval lower limit; IC, information component; IC_025_: 95% confidence interval of IC；MHRA proportional reporting ratio (PRR) method with the specific criteria set by the Medicines and Healthcare products Regulatory Agency; DEM/ IME：Designated Medical Events/ Important Medical Events.

**Supplementary Table 7** Signal strength for docetaxel-nintedanib combination at the preferred term level and the clinical priority assessing results

| Preferred term | Cases (n) | ROR(95%CI) | IC(IC_025_) | MHRA | Death (n) | DEM/ IME | Priority level (score) | BH-FDR | *Bonferroni*_*p* |
| --- | --- | --- | --- | --- | --- | --- | --- | --- | --- |
| Mucosal infection | 3 | 343.76 (109.66-1077.56) | 8.40 (0.53) | 343.31 (1005.78) | 0 | NA | Low(2) | p<0.001 | p<0.001 |
| Metastases to adrenals | 3 | 90.15 (28.97-280.47) | 6.49 (0.50) | 90.03 (262.89) | 0 | IME | Moderate(3) | p<0.001 | p<0.001 |
| Metastases to meninges | 4 | 46.64 (17.47-124.52) | 5.54 (0.91) | 63.23 (671.48) | 3 | IME | Moderate(4) | p<0.001 | p<0.001 |
| Metastases to central nervous system | 18 | 38.24 (24.04-60.83) | 5.24 (3.02) | 46.56 (177.9) | 4 | IME | Moderate(5) | p<0.001 | p<0.001 |
| Idiopathic pulmonary fibrosis | 6 | 37.27 (16.71-83.11) | 5.21 (1.50) | 37.95 (646.36) | 4 | IME | Moderate(3) | p<0.001 | p<0.001 |
| Metastases to peritoneum | 3 | 31.23 (10.06-97.00) | 4.96 (0.42) | 37.18 (210.82) | 3 | IME | Moderate(4) | p<0.001 | p<0.001 |
| Portal vein thrombosis | 3 | 28.43 (9.16-88.29) | 4.83 (0.41) | 31.19 (87.54) | 1 | IME | Moderate(4) | p<0.001 | p<0.001 |
| Peripheral sensory neuropathy | 6 | 28.27 (12.68-63.03) | 4.82 (1.44) | 28.40 (79.18) | 3 | IME | Moderate(5) | p<0.001 | p<0.001 |
| Neutropenic sepsis | 7 | 27.73 (13.20-58.27) | 4.79 (1.65) | 28.20 (157.19) | 0 | NA | Low(2) | p<0.001 | p<0.001 |
| Polyneuropathy | 12 | 26.77 (15.18-47.23) | 4.73 (2.36) | 27.65 (179.57) | 1 | DEM | Moderate(4) | p<0.001 | p<0.001 |
| Febrile neutropenia | 55 | 22.98 (17.59-30.04) | 4.49 (3.63) | 26.64 (295.78) | 7 | IME | Moderate(5) | p<0.001 | p<0.001 |
| Gastrointestinal toxicity | 4 | 22.88 (8.57-61.04) | 4.51 (0.80) | 22.46 (1127.59) | 14 | DEM | High(6) | p<0.001 | p<0.001 |
| Metastases to lung | 9 | 20.66 (10.73-39.77) | 4.36 (1.88) | 22.84 (83.44) | 2 | NA | Moderate(3) | p<0.001 | p<0.001 |
| Metastases to lymph nodes | 5 | 20.29 (8.43-48.83) | 4.34 (1.09) | 20.58 (167.52) | 0 | IME | Moderate(3) | p<0.001 | p<0.001 |
| Hepatic lesion | 3 | 19.17 (6.17-59.51) | 4.26 (0.34) | 20.25 (91.42) | 0 | IME | Moderate(3) | p<0.001 | p<0.001 |
| Metastases to liver | 12 | 18.43 (10.45-32.52) | 4.20 (2.17) | 19.14 (51.54) | 0 | NA | Low(2) | p<0.001 | p<0.001 |
| Mucosal inflammation | 16 | 17.55 (10.73-28.70) | 4.12 (2.44) | 18.34 (196.65) | 1 | IME | Moderate(3) | p<0.001 | p<0.001 |
| Haematotoxicity | 6 | 17.39 (7.80-38.75) | 4.12 (1.29) | 17.44 (247.75) | 4 | NA | Moderate(3) | p<0.001 | p<0.001 |
| Hiccups | 4 | 14.64 (5.49-39.07) | 3.87 (0.68) | 17.34 (92.34) | 3 | NA | Moderate(3) | p<0.001 | p<0.001 |
| Gamma-glutamyltransferase increased | 9 | 14.58 (7.58-28.07) | 3.86 (1.71) | 14.62 (50.73) | 1 | NA | Moderate(3) | p<0.001 | p<0.001 |
| Dermatitis acneiform | 3 | 13.82 (4.45-42.91) | 3.79 (0.27) | 14.53 (113.34) | 0 | NA | Low(2) | p<0.001 | p<0.001 |
| Transaminases increased | 11 | 13.66 (7.56-24.71) | 3.77 (1.89) | 13.81 (35.61) | 1 | NA | Moderate(3) | p<0.001 | p<0.001 |
| Pneumonitis | 14 | 13.60 (8.04-23.01) | 3.76 (2.13) | 13.6 (128.41) | 2 | NA | Low(2) | p<0.001 | p<0.001 |
| Neutropenia | 68 | 13.36 (10.5-17.01) | 3.70 (3.12) | 13.52 (162.33) | 7 | IME | Moderate(4) | p<0.001 | p<0.001 |
| Large intestine perforation | 3 | 12.72 (4.10-39.47) | 3.67 (0.25) | 13.00 (754.43) | 8 | IME | Moderate(4) | p<0.001 | p<0.001 |
| Stomatitis | 29 | 12.42 (8.61-17.92) | 3.62 (2.63) | 12.70 (32.32) | 1 | IME | Moderate(4) | p<0.001 | p<0.001 |
| Aspartate aminotransferase increased | 18 | 12.33 (7.76-19.61) | 3.61 (2.28) | 12.28 (300.49) | 3 | NA | Moderate(3) | p<0.001 | p<0.001 |
| Oesophagitis | 4 | 12.01 (4.50-32.03) | 3.58 (0.61) | 12.24 (185.88) | 2 | NA | Low(2) | p<0.001 | p<0.001 |
| Neutrophil count decreased | 18 | 12.00 (7.55-19.08) | 3.57 (2.25) | 11.99 (40.27) | 2 | NA | Moderate(3) | p<0.001 | p<0.001 |
| Metastasis | 3 | 11.45 (3.69-35.54) | 3.51 (0.22) | 11.91 (179.97) | 4 | NA | Low(2) | p<0.001 | p<0.001 |
| Haemoptysis | 11 | 10.99 (6.08-19.87) | 3.45 (1.74) | 11.44 (28.56) | 1 | IME | Moderate(4) | p<0.001 | p<0.001 |
| Atelectasis | 3 | 10.94 (3.52-33.95) | 3.45 (0.21) | 10.94 (99.34) | 5 | NA | Moderate(3) | p<0.001 | p<0.001 |
| Intestinal perforation | 4 | 10.27 (3.85-27.39) | 3.36 (0.55) | 10.93 (27.04) | 0 | NA | Low(2) | p<0.001 | p<0.001 |
| Alanine aminotransferase increased | 18 | 10.21 (6.42-16.24) | 3.34 (2.11) | 10.25 (33.38) | 4 | DEM | High(6) | p<0.001 | p<0.001 |
| Metastases to bone | 6 | 9.68 (4.34-21.58) | 3.27 (1.02) | 10.14 (148.37) | 2 | NA | Low(2) | p<0.001 | p<0.001 |
| Nail disorder | 3 | 9.28 (2.99-28.82) | 3.21 (0.15) | 9.66 (46.57) | 1 | IME | Moderate(3) | p<0.001 | p<0.001 |
| Leukopenia | 16 | 9.27 (5.67-15.17) | 3.20 (1.93) | 9.27 (22.14) | 1 | NA | Moderate(3) | p<0.001 | p<0.001 |
| Palmar-plantar erythrodysaesthesia syndrome | 8 | 9.08 (4.54-18.19) | 3.18 (1.29) | 9.22 (117.24) | 4 | IME | Moderate(4) | p<0.001 | p<0.001 |
| Hepatotoxicity | 7 | 8.54 (4.07-17.94) | 3.09 (1.11) | 9.06 (57.32) | 3 | NA | Moderate(3) | p<0.001 | p<0.001 |
| General physical health deterioration | 33 | 8.04 (5.70-11.34) | 2.99 (2.22) | 7.94 (200.38) | 15 | NA | Moderate(4) | p<0.001 | p<0.001 |
| Diarrhoea | 175 | 7.57 (6.49-8.83) | 2.82 (2.55) | 7.07 (922.06) | 39 | NA | Moderate(3) | p<0.001 | p<0.001 |
| Pulmonary embolism | 21 | 7.19 (4.68-11.06) | 2.84 (1.86) | 7.14 (110.95) | 6 | IME | Moderate(4) | p<0.001 | p<0.001 |
| Blood lactate dehydrogenase increased | 3 | 6.71 (2.16-20.82) | 2.74 (0.02) | 6.70 (14.55) | 1 | NA | Moderate(3) | 0.003 | 0.169 |
| White blood cell count decreased | 27 | 6.63 (4.54-9.70) | 2.72 (1.90) | 6.57 (127.66) | 6 | NA | Moderate(3) | p<0.001 | p<0.001 |
| Sepsis | 25 | 6.37 (4.29-9.45) | 2.66 (1.82) | 6.31 (111.9) | 17 | IME | High(6) | p<0.001 | p<0.001 |
| Hypokalaemia | 10 | 6.29 (3.38-11.71) | 2.65 (1.21) | 6.27 (44.31) | 1 | IME | Moderate(3) | p<0.001 | p<0.001 |
| Pleural effusion | 13 | 6.24 (3.62-10.77) | 2.64 (1.40) | 6.21 (56.91) | 6 | NA | Moderate(3) | p<0.001 | p<0.001 |
| Agranulocytosis | 4 | 6.21 (2.33-16.58) | 2.63 (0.31) | 6.21 (17.47) | 1 | DEM | Moderate(5) | p<0.001 | p<0.001 |
| Liver function test increased | 6 | 6.15 (2.76-13.71) | 2.62 (0.73) | 6.14 (25.81) | 0 | NA | Low(2) | p<0.001 | p<0.001 |
| Blood bilirubin increased | 5 | 6.12 (2.54-14.71) | 2.61 (0.54) | 6.10 (21.34) | 2 | NA | Moderate(3) | p<0.001 | p<0.001 |
| Blood alkaline phosphatase increased | 4 | 6.07 (2.28-16.19) | 2.60 (0.30) | 6.06 (16.91) | 0 | NA | Low(2) | p<0.001 | p<0.001 |
| C-reactive protein increased | 7 | 5.27 (2.51-11.08) | 2.39 (0.76) | 5.26 (24.16) | 1 | NA | Low(2) | p<0.001 | p<0.001 |
| Skin lesion | 5 | 5.01 (2.08-12.04) | 2.32 (0.40) | 5.00(15.99) | 2 | NA | Moderate(3) | p<0.001 | p<0.001 |
| Pericardial effusion | 4 | 5.01 (1.88-13.36) | 2.32 (0.18) | 5.00(12.81) | 2 | IME | Moderate(4) | 0.001 | 0.197 |
| Jaundice | 4 | 4.98 (1.87-13.29) | 2.31 (0.18) | 4.98 (12.71) | 1 | NA | Moderate(3) | 0.003 | 0.206 |
| Deep vein thrombosis | 9 | 4.58 (2.38-8.82) | 2.19 (0.84) | 4.57 (25.09) | 1 | IME | Moderate(3) | p<0.001 | p<0.001 |
| Anaemia | 30 | 4.52 (3.16-6.49) | 2.16 (1.49) | 4.48 (81.28) | 9 | NA | Moderate(4) | p<0.001 | p<0.001 |
| Hyponatraemia | 8 | 4.15 (2.07-8.30) | 2.05 (0.65) | 4.14 (19.03) | 3 | NA | Moderate(3) | p<0.001 | p<0.001 |
| Respiratory failure | 10 | 4.14 (2.23-7.71) | 2.05 (0.81) | 4.13 (23.74) | 8 | IME | Moderate(5) | p<0.001 | p<0.001 |
| Lymphadenopathy | 5 | 4.07 (1.69-9.79) | 2.02 (0.25) | 4.07 (11.56) | 0 | NA | Low(2) | 0.003 | 0.248 |
| Liver disorder | 6 | 3.95 (1.77-8.81) | 1.98 (0.38) | 3.95 (13.21) | 3 | NA | Moderate(3) | 0.001 | 0.097 |
| Decreased appetite | 32 | 3.63 (2.56-5.15) | 1.85 (1.23) | 3.60 (60.2) | 11 | NA | Moderate(4) | p<0.001 | p<0.001 |
| Colitis | 5 | 3.53 (1.47-8.48) | 1.82 (0.13) | 3.52 (9.03) | 1 | IME | Moderate(3) | 0.01 | 0.759 |
| Vomiting | 54 | 3.36 (2.56-4.40) | 1.72 (1.27) | 3.30 (87.22) | 10 | NA | Moderate(3) | p<0.001 | p<0.001 |
| Epistaxis | 8 | 2.83 (1.41-5.66) | 1.50 (0.27) | 2.82 (9.42) | 2 | NA | Moderate(3) | p<0.001 | p<0.001 |
| Alopecia | 23 | 2.81 (1.87-4.24) | 1.48 (0.79) | 2.79 (26.59) | 3 | NA | Moderate(3) | 0.006 | 0.435 |
| Blood creatinine increased | 6 | 2.80 (1.26-6.24) | 1.48 (0.06) | 2.80 (6.93) | 1 | NA | Low(2) | p<0.001 | p<0.001 |
| Neuropathy peripheral | 10 | 2.72 (1.46-5.06) | 1.44 (0.36) | 2.71 (10.83) | 0 | IME | Moderate(3) | 0.022 | 1 |
| Nausea | 71 | 2.55 (2.02-3.23) | 1.33 (0.95) | 2.51 (65.03) | 15 | NA | Moderate(3) | 0.003 | 0.190 |
| Pneumonia | 32 | 2.53 (1.79-3.59) | 1.33 (0.76) | 2.51 (29.3) | 14 | IME | Moderate(5) | p<0.001 | p<0.001 |
| Oedema peripheral | 8 | 2.48 (1.24-4.96) | 1.31 (0.12) | 2.47 (7.03) | 0 | NA | Low(2) | p<0.001 | p<0.001 |
| Dehydration | 11 | 2.45 (1.36-4.43) | 1.29 (0.29) | 2.44 (9.4) | 2 | NA | Low(2) | 0.018 | 1 |
| Asthenia | 32 | 2.37 (1.68-3.37) | 1.24 (0.67) | 2.36 (25.11) | 7 | NA | Moderate(3) | 0.005 | 0.362 |
| Paraesthesia | 12 | 2.16 (1.23-3.82) | 1.11 (0.18) | 2.16 (7.48) | 1 | NA | Low(2) | p<0.001 | p<0.001 |
| Pyrexia | 23 | 1.88 (1.25-2.84) | 0.91 (0.26) | 1.88 (9.45) | 5 | NA | Low(2) | 0.012 | 0.909 |
| Dyspnoea | 37 | 1.80 (1.30-2.49) | 0.84 (0.33) | 1.78 (12.88) | 16 | NA | Moderate(3) | 0.004 | 0.264 |
| Fatigue | 50 | 1.67 (1.26-2.21) | 0.73 (0.30) | 1.65 (13.09) | 12 | NA | Low(2) | p<0.001 | p<0.001 |

Abbreviations: SOC, system organ class; RORL, ROR 95% confidence interval lower limit; IC, information component; IC_025_: 95% confidence interval of IC；MHRA proportional reporting ratio (PRR) method with the specific criteria set by the Medicines and Healthcare products Regulatory Agency; DEM/ IME：Designated Medical Events/ Important Medical Events.

**Supplementary Table 8** Signal strength of reports of nintedanib at the preferred term and the clinical priority assessing results

| Preferred term | Cases (n) | ROR(95%CI) | IC(IC_025_) | MHRA | Death (n) | DEM/ IME | Priority level (score) | BH-FDR | *Bonferroni*_*p* |
| --- | --- | --- | --- | --- | --- | --- | --- | --- | --- |
| Cholangitis infective | 6 | 448.92 ( 195.85-1029.01 ) | 8.71 ( 1.65 ) | 447.86 ( 2494.83 ) | 3 | IME | Moderate(4) | p<0.001 | p<0.001 |
| Thrombophlebitis migrans | 3 | 177.22 ( 56.21-558.76 ) | 7.43 ( 0.5 ) | 177.01 ( 510.47 ) | 1 | IME | Moderate(4) | p<0.001 | p<0.001 |
| Biliary tract infection | 3 | 86.15 ( 27.55-269.39 ) | 6.41 ( 0.49 ) | 86.05 ( 248.72 ) | 1 | IME | Moderate(4) | p<0.001 | p<0.001 |
| Cholangitis | 15 | 68.94 ( 41.38-114.84 ) | 6.08 ( 2.98 ) | 68.53 ( 987.38 ) | 6 | IME | Moderate(4) | p<0.001 | p<0.001 |
| Enterocolitis | 13 | 68.25 ( 39.46-118.06 ) | 6.07 ( 2.77 ) | 67.91 ( 847.78 ) | 2 | IME | Moderate(3) | p<0.001 | p<0.001 |
| Metastases to peritoneum | 5 | 63.47 ( 26.28-153.31 ) | 5.97 ( 1.29 ) | 63.35 ( 303.72 ) | 2 | IME | Moderate(4) | p<0.001 | p<0.001 |
| Liver abscess | 6 | 56.71 ( 25.36-126.82 ) | 5.81 ( 1.56 ) | 56.58 ( 324.63 ) | 2 | IME | Moderate(4) | p<0.001 | p<0.001 |
| Myelosuppression | 17 | 34.31 ( 21.27-55.35 ) | 5.08 ( 2.9 ) | 34.08 ( 543.06 ) | 8 | IME | Moderate(4) | p<0.001 | p<0.001 |
| Haematotoxicity | 7 | 31.76 ( 15.1-66.82 ) | 4.98 ( 1.69 ) | 31.68 ( 206.93 ) | 1 | NA | Low(2) | p<0.001 | p<0.001 |
| Klebsiella infection | 5 | 31.1 ( 12.9-74.95 ) | 4.95 ( 1.19 ) | 31.04 ( 144.64 ) | 2 | NA | Moderate(3) | p<0.001 | p<0.001 |
| Portal vein thrombosis | 3 | 30.65 ( 9.85-95.38 ) | 4.93 ( 0.42 ) | 30.62 ( 85.54 ) | 1 | IME | Moderate(4) | p<0.001 | p<0.001 |
| Gastrointestinal toxicity | 4 | 29.48 ( 11.03-78.78 ) | 4.87 ( 0.84 ) | 29.43 ( 109.35 ) | 1 | NA | Moderate(3) | p<0.001 | p<0.001 |
| Peripheral sensory neuropathy | 5 | 27.49 ( 11.41-66.23 ) | 4.77 ( 1.16 ) | 27.44 ( 126.82 ) | 0 | NA | Low(2) | p<0.001 | p<0.001 |
| Metastases to lymph nodes | 6 | 27.46 ( 12.3-61.28 ) | 4.77 ( 1.43 ) | 27.39 ( 151.92 ) | 0 | IME | Moderate(3) | p<0.001 | p<0.001 |
| Jaundice cholestatic | 3 | 23.92 ( 7.69-74.37 ) | 4.57 ( 0.38 ) | 23.89 ( 65.54 ) | 1 | IME | Moderate(4) | p<0.001 | p<0.001 |
| Neutrophil count decreased | 31 | 23.14 ( 16.22-32.99 ) | 4.51 ( 3.25 ) | 22.86 ( 646.1 ) | 10 | NA | Moderate(4) | p<0.001 | p<0.001 |
| Hyperammonaemia | 4 | 20.61 ( 7.72-55.06 ) | 4.36 ( 0.77 ) | 20.58 ( 74.28 ) | 0 | NA | Low(2) | p<0.001 | p<0.001 |
| Ascites | 19 | 18.28 ( 11.63-28.72 ) | 4.18 ( 2.64 ) | 18.15 ( 307.05 ) | 3 | IME | Moderate(3) | p<0.001 | p<0.001 |
| Pulmonary toxicity | 3 | 12.92 ( 4.16-40.14 ) | 3.69 ( 0.25 ) | 12.91 ( 32.89 ) | 0 | IME | Moderate(3) | p<0.001 | p<0.001 |
| Metastases to liver | 8 | 11.62 ( 5.8-23.29 ) | 3.53 ( 1.45 ) | 11.59 ( 77.29 ) | 3 | IME | Moderate(4) | p<0.001 | p<0.001 |
| Malnutrition | 4 | 10.08 ( 3.78-26.9 ) | 3.33 ( 0.55 ) | 10.07 ( 32.61 ) | 0 | NA | Low(2) | p<0.001 | p<0.001 |
| Blood bilirubin increased | 8 | 9.83 ( 4.91-19.69 ) | 3.29 ( 1.34 ) | 9.8 ( 63.14 ) | 3 | NA | Moderate(3) | p<0.001 | p<0.001 |
| Leukopenia | 15 | 9.13 ( 5.49-15.17 ) | 3.18 ( 1.87 ) | 9.08 ( 107.72 ) | 3 | IME | Moderate(3) | p<0.001 | p<0.001 |
| Mucosal inflammation | 7 | 8.64 ( 4.11-18.15 ) | 3.11 ( 1.12 ) | 8.62 ( 47.09 ) | 1 | NA | Low(2) | p<0.001 | p<0.001 |
| Cholecystitis | 3 | 8.33 ( 2.68-25.86 ) | 3.05 ( 0.11 ) | 8.32 ( 19.3 ) | 0 | IME | Moderate(3) | p<0.001 | 0.024 |
| Urosepsis | 3 | 7.88 ( 2.54-24.48 ) | 2.98 ( 0.09 ) | 7.88 ( 17.99 ) | 0 | IME | Moderate(3) | p<0.001 | 0.040 |
| Neutropenia | 48 | 7.53 ( 5.66-10.02 ) | 2.89 ( 2.29 ) | 7.41 ( 266.46 ) | 13 | IME | Moderate(5) | p<0.001 | p<0.001 |
| Metastases to bone | 5 | 7.41 ( 3.08-17.82 ) | 2.88 ( 0.66 ) | 7.39 ( 27.62 ) | 1 | IME | Moderate(3) | p<0.001 | p<0.001 |
| Cytopenia | 3 | 6.6 ( 2.13-20.5 ) | 2.72 ( 0.01 ) | 6.6 ( 14.24 ) | 0 | IME | Moderate(3) | 0.003 | 0.160 |
| Intestinal obstruction | 8 | 6.53 ( 3.26-13.08 ) | 2.7 ( 1.05 ) | 6.52 ( 37.33 ) | 1 | IME | Moderate(3) | p<0.001 | p<0.001 |
| Cerebral infarction | 5 | 6.26 ( 2.6-15.05 ) | 2.64 ( 0.56 ) | 6.25 ( 22.02 ) | 2 | IME | Moderate(4) | p<0.001 | 0.002 |
| Septic shock | 9 | 6.17 ( 3.21-11.88 ) | 2.62 ( 1.11 ) | 6.15 ( 38.82 ) | 7 | IME | Moderate(5) | p<0.001 | p<0.001 |
| Diarrhoea | 153 | 6.02 ( 5.11-7.09 ) | 2.51 ( 2.23 ) | 5.72 ( 601.65 ) | 30 | NA | Moderate(3) | p<0.001 | p<0.001 |
| Decreased appetite | 48 | 5.17 ( 3.89-6.88 ) | 2.35 ( 1.81 ) | 5.09 ( 158.25 ) | 11 | NA | Moderate(3) | p<0.001 | p<0.001 |
| Pneumonitis | 5 | 5.04 ( 2.09-12.11 ) | 2.33 ( 0.41 ) | 5.03 ( 16.12 ) | 0 | IME | Moderate(3) | p<0.001 | 0.029 |
| Febrile neutropenia | 11 | 5.01 ( 2.77-9.07 ) | 2.32 ( 1.07 ) | 5 ( 35.15 ) | 3 | DME | Moderate(5) | p<0.001 | p<0.001 |
| Hepatic failure | 5 | 4.92 ( 2.05-11.84 ) | 2.3 ( 0.39 ) | 4.91 ( 15.58 ) | 4 | DME | High(6) | p<0.001 | 0.037 |
| Oral pain | 4 | 4.89 ( 1.83-13.03 ) | 2.29 ( 0.17 ) | 4.88 ( 12.33 ) | 0 | NA | Low(2) | 0.004 | 0.203 |
| Hypokalaemia | 9 | 4.49 ( 2.33-8.64 ) | 2.16 ( 0.82 ) | 4.48 ( 24.31 ) | 3 | IME | Moderate(4) | p<0.001 | p<0.001 |
| Jaundice | 4 | 4.48 ( 1.68-11.95 ) | 2.16 ( 0.11 ) | 4.47 ( 10.78 ) | 3 | NA | Moderate(4) | 0.007 | 0.387 |
| Stomatitis | 11 | 4.36 ( 2.41-7.88 ) | 2.12 ( 0.93 ) | 4.34 ( 28.29 ) | 1 | NA | Low(2) | p<0.001 | p<0.001 |
| White blood cell count decreased | 16 | 4.32 ( 2.64-7.06 ) | 2.1 ( 1.14 ) | 4.3 ( 40.5 ) | 3 | NA | Low(2) | p<0.001 | p<0.001 |
| Hypophagia | 4 | 4.05 ( 1.52-10.79 ) | 2.01 ( 0.04 ) | 4.04 ( 9.16 ) | 1 | NA | Moderate(3) | 0.013 | 0.768 |
| Blood potassium decreased | 5 | 4.05 ( 1.68-9.73 ) | 2.01 ( 0.24 ) | 4.04 ( 11.43 ) | 0 | NA | Low(2) | 0.004 | 0.222 |
| Anaemia | 27 | 3.77 ( 2.58-5.51 ) | 1.9 ( 1.22 ) | 3.74 ( 54.31 ) | 7 | NA | Moderate(4) | p<0.001 | p<0.001 |
| Pulmonary embolism | 13 | 3.77 ( 2.18-6.5 ) | 1.91 ( 0.87 ) | 3.75 ( 26.27 ) | 5 | IME | Moderate(4) | p<0.001 | p<0.001 |
| Neuropathy peripheral | 14 | 3.68 ( 2.18-6.23 ) | 1.87 ( 0.89 ) | 3.67 ( 27.18 ) | 0 | IME | Moderate(3) | p<0.001 | p<0.001 |
| Thrombocytopenia | 14 | 3.66 ( 2.16-6.19 ) | 1.87 ( 0.88 ) | 3.65 ( 26.92 ) | 2 | IME | Moderate(3) | p<0.001 | p<0.001 |
| Colitis | 5 | 3.44 ( 1.43-8.28 ) | 1.78 ( 0.11 ) | 3.44 ( 8.65 ) | 0 | IME | Moderate(3) | 0.013 | 0.760 |
| Multiple organ dysfunction syndrome | 5 | 3.3 ( 1.37-7.94 ) | 1.72 ( 0.07 ) | 3.3 ( 8 ) | 5 | IME | Moderate(5) | 0.017 | 1.000 |
| Aspartate aminotransferase increased | 5 | 3.28 ( 1.37-7.9 ) | 1.71 ( 0.07 ) | 3.28 ( 7.93 ) | 1 | NA | Low(2) | 0.017 | 1.000 |
| Vomiting | 56 | 3.16 ( 2.43-4.12 ) | 1.64 ( 1.2 ) | 3.11 ( 80.92 ) | 7 | NA | Moderate(3) | p<0.001 | p<0.001 |
| Pancreatitis | 6 | 3.16 ( 1.42-7.04 ) | 1.66 ( 0.18 ) | 3.15 ( 8.82 ) | 1 | DME | Moderate(4) | 0.010 | 0.601 |
| Sepsis | 12 | 3.06 ( 1.73-5.39 ) | 1.61 ( 0.59 ) | 3.05 ( 16.51 ) | 4 | IME | Moderate(4) | p<0.001 | 0.009 |
| Death | 104 | 2.76 ( 2.27-3.36 ) | 1.42 ( 1.11 ) | 2.69 ( 111.69 ) | 104 | IME | High(6) | p<0.001 | p<0.001 |
| Herpes zoster | 6 | 2.7 ( 1.21-6.01 ) | 1.43 ( 0.02 ) | 2.69 ( 6.39 ) | 0 | NA | Low(2) | 0.029 | 1.000 |
| Platelet count decreased | 11 | 2.7 ( 1.5-4.89 ) | 1.43 ( 0.4 ) | 2.7 ( 11.76 ) | 2 | NA | Low(2) | 0.002 | 0.097 |
| Pyrexia | 31 | 2.45 ( 1.72-3.5 ) | 1.28 ( 0.71 ) | 2.43 ( 26.34 ) | 4 | NA | Moderate(3) | p<0.001 | p<0.001 |
| Nausea | 71 | 2.38 ( 1.88-3.01 ) | 1.23 ( 0.85 ) | 2.34 ( 55.09 ) | 11 | NA | Moderate(3) | p<0.001 | p<0.001 |
| Abdominal pain | 20 | 2.32 ( 1.49-3.6 ) | 1.21 ( 0.49 ) | 2.31 ( 14.9 ) | 7 | NA | Moderate(3) | p<0.001 | 0.015 |
| Dehydration | 10 | 2.04 ( 1.09-3.79 ) | 1.02 ( 0.02 ) | 2.03 ( 5.26 ) | 3 | NA | Moderate(3) | 0.039 | 1.000 |
| Malaise | 37 | 1.95 ( 1.41-2.7 ) | 0.95 ( 0.44 ) | 1.93 ( 16.82 ) | 10 | NA | Moderate(3) | p<0.001 | 0.004 |
| Back pain | 17 | 1.84 ( 1.14-2.97 ) | 0.88 ( 0.13 ) | 1.84 ( 6.51 ) | 0 | NA | Low(1) | 0.018 | 1.000 |
| Constipation | 17 | 1.79 ( 1.11-2.89 ) | 0.84 ( 0.09 ) | 1.79 ( 5.92 ) | 2 | NA | Low(1) | 0.024 | 1.000 |
| Fatigue | 49 | 1.52 ( 1.15-2.02 ) | 0.6 ( 0.17 ) | 1.51 ( 8.57 ) | 15 | NA | Moderate(3) | 0.005 | 0.298 |

Abbreviations: SOC, system organ class; RORL, ROR 95% confidence interval lower limit; IC, information component; IC_025_: 95% confidence interval of IC；MHRA proportional reporting ratio (PRR) method with the specific criteria set by the Medicines and Healthcare products Regulatory Agency; DEM/ IME：Designated Medical Events/ Important Medical Events.

**Supplementary Table 9** Signal strength for docetaxel at the preferred term level (Excluding lawyer)

| Preferred term | Cases (n) | ROR(95%CI) | IC(IC_025_) | Signal Retained |
| --- | --- | --- | --- | --- |
| Psychological trauma | N/A | N/A | N/A | No |
| Madarosis | 6 | 7.23 ( 3.09 - 16.87 ) | 2.71 ( 0.7 ) | yes |
| Dacryostenosis acquired | 12 | 41.68 ( 19.9 - 87.29 ) | 4.63 ( 2.16 ) | yes |
| Ecthyma | 4 | 236.01 ( 26.38 - 2111.82 ) | 5.58 ( 0.44 ) | yes |
| Pneumonia salmonella | 4 | 236.01 ( 26.38 - 2111.82 ) | 5.58 ( 0.44 ) | yes |
| Alopecia | 384 | 12.04 ( 10.78 - 13.46 ) | 3.31 ( 3.11 ) | yes |
| Hair injury | N/A | N/A | N/A | No |
| Deformity | 5 | 98.35 ( 23.5 - 411.58 ) | 5.23 ( 0.86 ) | yes |
| Emotional distress | 101 | 79.08 ( 58.69 - 106.56 ) | 5.1 ( 4.32 ) | yes |
| Ischaemic enteritis | 4 | 78.67 ( 17.6 - 351.55 ) | 5.1 ( 0.5 ) | yes |
| Orthostatic intolerance | 4 | 78.67 ( 17.6 - 351.55 ) | 5.1 ( 0.5 ) | yes |
| Osmotic demyelination syndrome | 4 | 78.67 ( 17.6 - 351.55 ) | 5.1 ( 0.5 ) | yes |
| Hypereosinophilic syndrome | 11 | 72.16 ( 29.9 - 174.17 ) | 5.04 ( 2.11 ) | yes |
| Mucosal infection | 12 | 59.04 ( 26.52 - 131.45 ) | 4.91 ( 2.21 ) | yes |
| Salmonella sepsis | 4 | 78.67 ( 17.6 - 351.55 ) | 5.1 ( 0.5 ) | yes |
| Splenic vein thrombosis | 4 | 33.72 ( 9.87 - 115.19 ) | 4.45 ( 0.52 ) | yes |
| Bipolar disorder | N/A | N/A | N/A | No |
| Lacrimation increased | 30 | 6.79 ( 4.65 - 9.92 ) | 2.63 ( 1.86 ) | yes |
| Major depression | N/A | N/A | N/A | No |
| Mediastinitis | 8 | 27.78 ( 11.98 - 64.37 ) | 4.26 ( 1.53 ) | yes |
| Depression | N/A | N/A | N/A |  |
| Necrotising fasciitis | 10 | 26.83 ( 12.7 - 56.68 ) | 4.23 ( 1.82 ) | yes |
| Coating in mouth | 4 | 26.22 ( 8.07 - 85.17 ) | 4.21 ( 0.52 ) | yes |
| Eye injury | N/A | N/A | N/A | No |
| Obstruction gastric | 9 | 26.56 ( 12.09 - 58.35 ) | 4.22 ( 1.68 ) | yes |
| Pathogen resistance | 10 | 21.86 ( 10.58 - 45.18 ) | 4.02 ( 1.76 ) | yes |
| Anxiety | 106 | 5.29 ( 4.33 - 6.46 ) | 2.29 ( 1.95 ) | yes |
| Hypoventilation | 6 | 19.67 ( 7.81 - 49.56 ) | 3.91 ( 1.07 ) | yes |
| Haemolytic uraemic syndrome | 11 | 18.04 ( 9.18 - 35.45 ) | 3.81 ( 1.81 ) | yes |
| Epidermolysis | 6 | 16.86 ( 6.8 - 41.78 ) | 3.74 ( 1.04 ) | yes |
| Neutropenic infection | 6 | 16.86 ( 6.8 - 41.78 ) | 3.74 ( 1.04 ) | yes |
| Onycholysis | 8 | 13.89 ( 6.43 - 30.01 ) | 3.51 ( 1.33 ) | yes |
| Atypical haemolytic uraemic syndrome | 5 | 16.39 ( 6.08 - 44.16 ) | 3.7 ( 0.78 ) | yes |
| Oesophageal fistula | 6 | 15.39 ( 6.27 - 37.81 ) | 3.63 ( 1.01 ) | yes |
| Personality change | 7 | 14.75 ( 6.44 - 33.78 ) | 3.58 ( 1.19 ) | yes |
| Blepharitis | 6 | 9.57 ( 4.04 - 22.68 ) | 3.07 ( 0.84 ) | yes |
| Traumatic lung injury | N/A | N/A | N/A | No |
| Emotional disorder | N/A | N/A | N/A | No |
| Venous thrombosis | 13 | 7.68 ( 4.31 - 13.68 ) | 2.79 ( 1.45 ) | yes |
| Mental disorder |  |  |  | No |
| Onychomadesis | 9 | 4.96 ( 2.51 - 9.8 ) | 2.22 ( 0.81 ) | yes |
| Stress | N/A | N/A | N/A | No |
| Polyneuropathy | 33 | 5.81 ( 4.06 - 8.31 ) | 2.42 ( 1.73 ) | yes |
| Agranulocytosis | 42 | 5.43 ( 3.95 - 7.45 ) | 2.33 ( 1.74 ) | yes |
| Peripheral sensory neuropathy | 26 | 5.51 ( 3.68 - 8.23 ) | 2.35 ( 1.57 ) | yes |
| Therapeutic response decreased | 23 | 5.29 ( 3.45 - 8.1 ) | 2.3 ( 1.46 ) | yes |
| Neutropenic sepsis | 36 | 5.32 ( 3.78 - 7.49 ) | 2.31 ( 1.66 ) | yes |
| White blood cell count decreased | N/A | N/A | N/A | No |
| Stomatitis | 140 | 4.26 ( 3.59 - 5.07 ) | 2 ( 1.72 ) | yes |
| Psychological trauma | N/A | N/A | N/A | No |
| Madarosis | 6 | 7.23 ( 3.09 - 16.87 ) | 2.71 ( 0.7 ) | yes |
| Hair disorder | N/A | N/A | N/A | No |
| Hair colour changes | N/A | N/A | N/A | No |
| Hair texture abnormal | N/A | N/A | N/A | No |
| Self esteem decreased | N/A | N/A | N/A | No |
| Post-traumatic stress disorder | N/A | N/A | N/A | No |
| Dacryostenosis acquired | 12 | 41.68 ( 19.9 - 87.29 ) | 4.63 ( 2.16 ) | yes |
| Ecthyma | 4 | 236.01 ( 26.38 - 2111.82 ) | 5.58 ( 0.44 ) | yes |
| Pneumonia salmonella | 4 | 236.01 ( 26.38 - 2111.82 ) | 5.58 ( 0.44 ) | yes |
| Alopecia | 384 | 12.04 ( 10.78 - 13.46 ) | 3.31 ( 3.11 ) | yes |
| Hair injury | N/A | N/A | N/A | No |
| Deformity | 5 | 98.35 ( 23.5 - 411.58 ) | 5.23 ( 0.86 ) | yes |
| Emotional distress | 101 | 79.08 ( 58.69 - 106.56 ) | 5.1 ( 4.32 ) | yes |
| Ischaemic enteritis | 4 | 78.67 ( 17.6 - 351.55 ) | 5.1 ( 0.5 ) | yes |
| Orthostatic intolerance | 4 | 78.67 ( 17.6 - 351.55 ) | 5.1 ( 0.5 ) | yes |
| Osmotic demyelination syndrome | 4 | 78.67 ( 17.6 - 351.55 ) | 5.1 ( 0.5 ) | yes |
| Hypereosinophilic syndrome | 11 | 72.16 ( 29.9 - 174.17 ) | 5.04 ( 2.11 ) | yes |
| Mucosal infection | 12 | 59.04 ( 26.52 - 131.45 ) | 4.91 ( 2.21 ) | yes |
| Salmonella sepsis | 4 | 78.67 ( 17.6 - 351.55 ) | 5.1 ( 0.5 ) | yes |
| Hair disorder | N/A | N/A | N/A | No |
| Hair colour changes | N/A | N/A | N/A | No |
| Hair texture abnormal | N/A | N/A | N/A | No |
| Self esteem decreased | N/A | N/A | N/A | No |
| Post-traumatic stress disorder | N/A | N/A | N/A | No |

**Supplementary Figure 1.** Difference risk signals of age subgroups in docetaxel-nintedanib combination.

**Supplementary Figure 2.** Difference risk signals of sex subgroups in docetaxel-nintedanib combination.

**Supplementary Figure 3.** Difference risk signals of age subgroups in docetaxel monotherapy.

**Supplementary Figure 4**. Difference risk signals of sex subgroups in docetaxel monotherapy.

**Supplementary Figure 5.** Paired ROR comparison of top 20 PTs with maximum absolute ROR shifts pre- and post-exclusion of lawyer-submitted reports

**Supplementary Figure 6.** (A). Cumulative incidence curves of ADR by age in docetaxel-nintedanib combination; (B). Cumulative incidence curves of ADR by sex in docetaxel-nintedanib combination; (C). Cumulative incidence curves of ADR by age in docetaxel; (D). Cumulative incidence curves of ADR by sex in docetaxel.
